# Mechanism of the Direct Reduction of Chromite Process as a Clean Ferrochrome Technology

**DOI:** 10.1021/acsengineeringau.3c00057

**Published:** 2023-12-01

**Authors:** Dogan Paktunc, Jason P. Coumans, David Carter, Nail Zagrtdenov, Dominique Duguay

**Affiliations:** CanmetMINING, Natural Resources Canada, 555 Booth Street, Ottawa, ON K1A 0G1, Canada

**Keywords:** ferrochrome, chromium, Cr−Fe carbide, slag, chromite, carbothermic, XANES, EXAFS

## Abstract

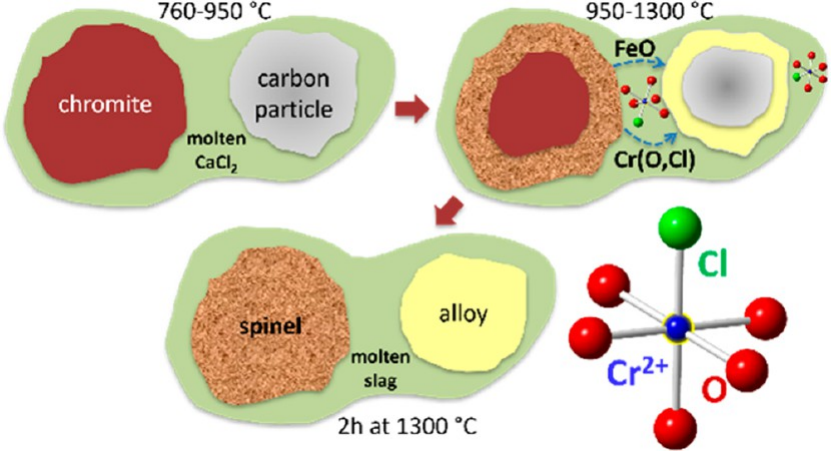

Direct reduction of chromite (DRC) is a promising alternative process for ferrochrome production with the potential to significantly reduce energy consumption and greenhouse gas emissions compared to conventional smelting. In DRC, chromium (Cr) and iron (Fe) from chromite ore incongruently dissolve into a molten salt, which facilitates mass transfer to a carbon (C) reductant where in situ metallization occurs. Consequently, ferrochrome is produced below the slag melting temperatures, achieving substantial energy savings relative to smelting. However, there are significant knowledge gaps in the kinetics, Cr solubility, speciation, and coordination environment which are critical to understanding the fundamental mechanisms of molten salt-assisted carbothermic reactions. To address these knowledge gaps, we performed pyrometallurgical experiments with variable temperature and residence times and analyzed the composition of chromite, ferrochrome, and slag products along with determining the speciation of Cr. Our results indicate that the DRC mechanism can be explained by the following sequential steps: (1) incongruent dissolution of chromite, (2) reduction of dissolved Cr in molten salt/slag, (3) transport of Cr and Fe species in molten media, and (4) reduction on C particles and metallization as Cr–Fe alloys. The discovery of four types of reduced Cr species in the slag indicates that the reduction of Cr^3+^ to Cr^2+^ and Cr^0^ occurred in the molten phase before metallization on solid carbon particles. Thermodynamically, the reduction of CrO(*l*) to Cr metal is more feasible at a lower temperature than it is for Cr_2_O_3_(*l*) corroborating the accelerated reduction efficiency of the DRC process.

## 1 Introduction

Ferrochrome is typically produced by the smelting of chromite ore in electric arc furnaces. Conventional smelting processes are energy intensive with variable energy consumptions from about 3 to 7 MWh per ton ferrochrome produced^1^ with the lower range belonging to most advanced technologies like closed submerged arc furnace with oxidized or prereduced pellet feeds and DC-arc with preheated feed. On average, the energy requirements are greater than 4 MWh/t.^2,3^ In addition, greenhouse gas emissions related to smelting in electric arc furnaces can exceed 10 t of CO_2_ per ton of Cr in ferrochrome produced.^1^ Energy efficient and low-carbon processes are critical for sustainability and competitiveness of the ferrochrome industry.

Molten salts offer unique properties of high dissolving power, thermal stability, inertia, and heat transfer^4^ which make them attractive as fluxes or catalysts in reduction reactions. Our earlier studies therefore investigated the potential use of several alkalis, cryolite (Na_3_AlF_6_) and CaCl_2_ as fluxes, which indicated that the carbothermic reduction of chromite at around 1300 °C is significantly accelerated.^5−10^ In the presence of NaOH as the flux, chromite ore is reduced to Cr_4.2–4.9_Fe_2.1–2.8_C_3_ with 85% Cr metallization after 2 h of reaction at 1300 °C.^5^ Similarly, addition of cryolite which is widely used as an electrolyte in the production of aluminum also accelerated the kinetics of chromite reduction and resulted in the formation of coarse ferrochrome alloy particles after 2 h of reduction at 1300 °C.^7^ Overall, the use of molten salts enables the formation of a liquid layer around chromite particles to allow the mass transport of reducible cations. In the case of CaCl_2_ flux, the direct reduction of chromite (DRC) process typically results in over 94% Cr and 100% Fe metallization in the form of discrete particles of ferrochrome that can measure several hundred microns.^8^ Pelletized feed ensures that low oxygen partial pressures less than about 10^–11^ atm can be attained locally in close proximity to chromite and less than about 10^–16^ atm near carbon particles.^9^ Due to the presence of solid carbon, a high *P*_CO_/*P*_CO_2__ of about 2000 and greater is maintained within pellets throughout the reduction reactions. This makes the pellets behave like mini reactors during the DRC process.

The DRC process is emerging as an alternate process for ferrochrome production with the potential of reducing energy consumption and greenhouse gas emissions. This process can be defined as the production of a metallic ferrochrome from chromite ore using a carbon reductant in the presence of a molten salt without melting the whole feed. It differs from smelting in electric arc furnaces where the whole feed is melted and ferrochrome separated from the slag by tapping of density segregated molten materials. In the case of DRC, final products are solid, and the density difference between alloy and slag products makes the separation and recovery of alloy possible by conventional gravity techniques.

The feed composed of chromite ore and CaCl_2_ flux mixed with stoichiometric amount of carbon is agglomerated and reduced at 1300 °C for 2 h. Ferrochrome is typically an M_7_C_3_ type carbide having an average composition reflecting the Cr/Fe ratio of the chromite ore. The product is water leached to separate the solids and remove CaCl_2_. The solids are liberated ferrochrome and slag particles measuring between about 35 and 200 μm, which are readily recovered through simple gravity separation techniques. The Cr/Fe ratio of the alloy concentrate is close to its original value in chromite, suggesting that Cr reduction was efficient with excellent Cr and Fe recoveries. Typical recovery figures are greater than 86% for alloy grades of 97% by mass. In addition to ferrochrome, the slag, which is largely a refractory spinel compound (i.e., MgAl_2_O_4_), can be considered as a saleable byproduct. The leachate from the water leaching is evaporated to recover CaCl_2_ which is then recirculated for reuse as a flux. Recoveries of more than 55 wt % of the original CaCl_2_ (anhydrous) in the feed is typical. Rotary kiln and rotating hearth reactors heated with natural gas are potential furnaces for the DRC process.

Technology development and demonstration of this process is well underway with prepilot studies involving large-scale testing, development of process flow diagrams and CFD modeling, placing the Technology Readiness Level (TRL) of this process at 7 meaning that the technology is proven and ready for piloting. Preliminary techno economic analyses indicate that the total production cost of the DRC process and greenhouse gas emissions are about 30% lower than those of the most advanced smelting technologies. Thus, the CaCl_2_-assisted direct reduction process is highly efficient and promising in terms of its cost and environmental performance with little waste produced.

As our working hypothesis, it is contemplated that the CaCl_2_-assisted DRC process involves incongruent dissolution of chromite in molten CaCl_2_ followed by in situ reduction and metallization of Fe and Cr species on solid carbon particles.^8−10^ There are, however, significant gaps in knowledge on the kinetics and mechanism of the DRC process, including the influence of speciation and solubility of Cr and molten slag chemistry on the transport of Cr from chromite to reduction sites. Developing a fundamental level understanding of the carbothermic reactions and evolution of Cr and Fe species during direct reduction is an essential prerequisite for addressing these knowledge gaps, optimizing the process parameters, and advancing the technological viability of the DRC process. To gain insights into these knowledge gaps and to test our working hypothesis, we conducted experiments to examine the influences of temperature and residence time on the composition of chromite, ferrochrome, and slag products, along with determining the speciation of Cr during direct reduction of chromite.

## 2 Materials and Methods

Pellets measuring 3 to 12 mm across were prepared from fine particulate materials composed of 67–69 wt % ore, 10–15 wt % petroleum coke (petcoke) as the reductant, and 13-21 wt % CaCl_2_ as the flux with and without a small amount of bentonite as a binder, as shown in Table 1. These compositions are based on optimization studies performed on typical Ring of Fire chromite ore (SI Table S1), made of 78 wt % chromite and 22 wt % clinochlore. The amount of the reductant is slightly greater than the stoichiometric carbon to maintain the partial pressures of CO/CO_2_ as per the Boudouard equilibrium. Ore and petcoke particles are in the −106 + 38 μm size range and CaCl_2_ as a fine powdered material. Petcoke has 99.8 wt % fixed carbon.

**Table 1:** Experimental Conditions and Design Considerations

exp#	feed^a^	*T* (°C)	*t* (min)^b^	experimental design consideration
1	67:13:20	800	15	clinochlore dissolution; beginning of incongruent dissolution of chromite
2	67:13:20	950	15	Fe reduction by CO
3	67:13:20	1000	5	Fe reduction by solid C
4	67:13:20	1100	5	onset of Cr reduction by solid carbon
5	67:13:20	1200	5	nearing end of Fe reduction
6	67:13:20	1200	15	increased endothermic Cr reduction
7	67:13:20	1200	30	Cr transport in slag
8	67:13:20	1300	1	onset of peak Cr reduction
9	67:13:20	1300	15	sustained Cr reduction with rate control by C diffusion across alloy
10	67:13:20	1300	120	complete reduction
11	69:10:21	1300	240	saturation of slag with respect to Cr in the absence of reductant
12	67:15:13:5	1300	180	increased silicate slag with Cr speciation

a Feed: mass proportions of ore, reductant and CaCl_2_ for experiments 1–11, and ore, reductant, CaCl_2_ and bentonite for experiment 12.

b *t*: dwell time in minutes at target reduction temperature.

Experimental design considerations included reduction temperatures varying from 800 to 1300 °C for reaction times variable from 1 to 240 min aiming to capture the evolution of slag, alloy and chromite compositions, and determine the solubility of Cr in slag melt (Table 1). In addition, an experiment was conducted at 1300 °C for a prolonged period of 4 h involving substoichiometric carbon to determine Cr saturation or its solubility limit and to test the changes in Cr species after the depletion of carbon. The absence of reductant would ensure that Cr in slag melt increases with continued dissolution of chromite, enabling changes in speciation of Cr following its dissolution from chromite. Experiments were carried out in a sealed vertical tube furnace (VTF) coupled to an infrared gas analyzer and a Netzsch thermogravimetry analyzer with differential scanning calorimetry (TGA-DSC) instrument coupled with a mass spectrometer. For the VTF, heating and cooling rates were 5 and 10 °C/min, respectively. For the TGA-DSC, heating and cooling rates were 10 and 20–50 °C/min, respectively. Grade 5.0 Argon at a flow rate of up to 1.0 L/min was used as needed to maintain an inert atmosphere. The CO content in the off-gas was converted to fluxes, normalized with respect to the ore content, and integrated to evaluate the extent of reduction.

Quantitative X-ray microanalyses were made by wavelength-dispersive spectrometry (WDS) using a JEOL JXA 8230 electron probe microanalyzer operated at an accelerating voltage of 20 kV, and a probe current of 20 to 60 nA. Counting times for discrete phase analyses ranged from 15 to 40 s on peak and background. Quantitative X-ray maps were collected with 0.1 to 0.3 μm steps and a 40 ms dwell time. Reported microanalyses with two decimal points are based on 3 sigma uncertainty with a confidence level of 99.73%. Partitioning of the measured Fe between divalent and trivalent species was based on stoichiometry. Carbon concentrations of the alloy phase were determined by difference which is consistent for M_7_C_3_ type carbides.

Phase quantities were determined by quantitative mineralogy techniques using a TESCAN Integrated Mineral Analyzer (TIMA) equipped with four silicon-drift energy-dispersive X-ray detectors. The analyses were performed at an accelerating voltage of 25 kV and a beam current of 5.5 nA using the high-resolution mapping mode with a step size of 0.5 μm.

Synchrotron-based X-ray absorption near edge structure (XANES) and extended X-ray absorption fine structure (EXAFS) spectroscopy experiments at the Cr *K*-edge were carried out at the insertion device beamlines, 20-ID and 13-IDE of the Advanced Photon Source, and I18 of the Diamond Light Source. Micro-XANES and micro-EXAFS spectra were collected using a 2 × 2.5 μm beam on epoxy impregnated pellets cut and polished as thin sections. Experiments at 20-ID utilized a confocal microchannel array optics providing 2–5 μm depth resolution and limiting detection of emissions to small volumes of materials at specified depths along the path of the focused beam through the sample. Micro-XRF maps and micro-XANES spectra collected at 13-IDE and I18 are subject to minor contamination from particles hidden below the surface. EXAFS spectra from the reference samples (CaCl_2_, CaCl_3_, Cr_2_O_3_, chromite, Cr_4.5_Fe_2.5_C_3_, Cr_3_C_2_, and Cr_23_C_6_) were collected at the bending magnet beamline, 20-BM of the Advanced Photon Source. Measurements were made on finely ground samples, spread onto tapes as monolayers. Samples were scanned four to five times in transmission and fluorescence modes using a beam size of 1 × 2 mm. Data reduction and analysis were done by Larch/ATHENA/ARTEMIS/FEFF^11,12^ and the least-squares fitting analyses of the XANES spectra were performed with LSFitXAFS.^13^ EXAFS data analysis considered the theoretical phase and amplitude functions generated in FEFF8.2.

Thermodynamics calculations were performed using equilibrium, reaction, and phase diagram modules of FactSage8.1.^14^ Databases included in the calculations are FSstel, Ftoxid, Ftsalt, and FactPS. Equilibrium calculations were performed using the same ore composition and mass proportions of ore, reductant, and flux used in experiments.

## 3 Results and Discussion

### 3.1 Reaction Kinetics

**Figure 1:**
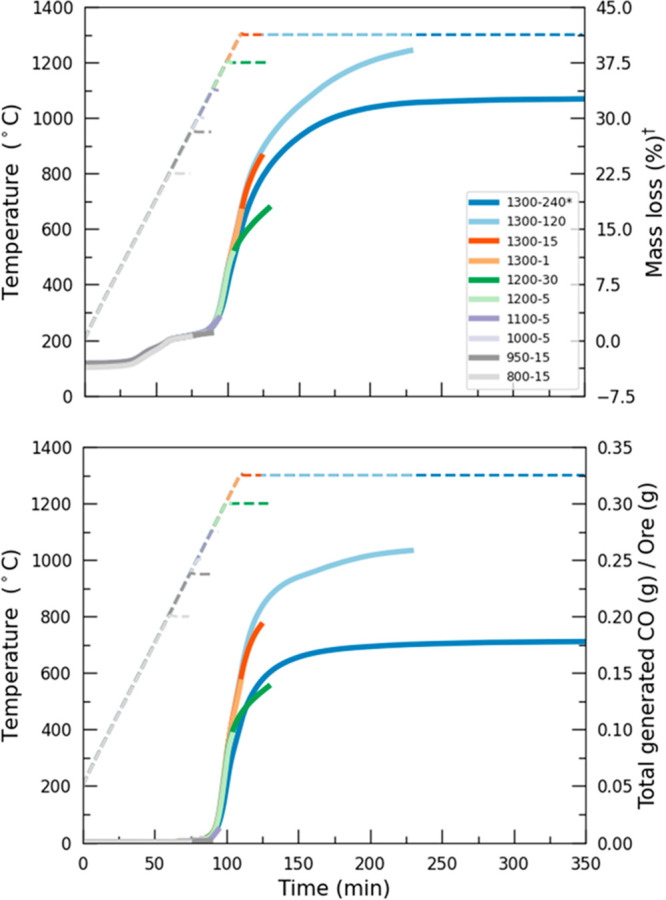
Thermogravimetry measurements and evolution of CO concentrations with time during experiments conducted over the 800–1300 °C range. Dashed lines represent temperature profiles, and solid lines are for mass losses (top) and cumulative CO generated (bottom).

Mass losses include loss of structural water from clinochlore at around 500 °C (3–4%) prior to CaCl_2_ melting at ∼760 °C (Figure 1). Following these events, mass losses corresponding to rapid increases in CO generation occur between 1000 and 1300 °C due to reduction events. The mass losses following melting of clinochlore are 2.5%, 11.0%, and 17.0% for experiments 1100-5, 1200-5 and 1300–1 respectively. The normalized CO generations are 0.015, 0.095, and 0.145 g CO per gram ore for experiments 1100-5, 1200-5 and 1300–1, respectively. Integration of the CO peak indicates that the bulk of the reduction takes place within about 1 h at 1300 °C. The reduction reactions appear to slow down between ∼1200 and 1300 °C, indicating the dominance of endothermic Cr reduction reactions and the effect of decreasing solid carbon. The cumulative CO generation is further slowed down after 1300 °C, possibly pointing toward a switch in the rate limiting processes. After 120 min at a dwell temperature of 1300 °C, most of the reductant particles have been consumed, and the total CO generated plateaus. At these conditions, mass loss and CO generation are 38.5% and 0.260 g of CO per gram of ore, respectively. Lower mass loss and CO generation observed for the experiment at 1300 °C, after 240 min (i.e., 31.5% and 0.165 g of CO per gram of ore) are attributable to the substoichiometric amount of C available for reduction reactions. These events are in accordance with the thermodynamic predictions of mass losses of about 1.8% at 900 °C, increasing to 3.9% at 1000 °C, 9.7% at 1100 °C and greater than about 20% at 1200 °C. There are gradual decreases in the equilibrium proportions of chromite from 55.7 to 44.5 wt % and solid carbon from 14.7 to 12.1 wt % across the temperature interval of 800 to 1100 °C. Accompanying these changes are gradual increases in the CO and alloy contents of the products (i.e., from near 0 to 4.9 wt % for CO and to 9 wt % for the alloy). The compositional changes in the equilibrium end-products are drastic in the 1100–1200 °C range and more gradual from 1200 to 1300 °C.

Measured phase quantities using TIMA indicate the presence of 1.8 wt % metallic Fe and 96 wt % chromite in the product resulted from reduction at 950 °C after 15 min (Experiment 2). These results corresponding to alloy/slag ratio of 0.02 indicate that equilibrium is not reached (Table 2). At 1200 °C after 30 min (Experiment 7), observed phase quantities are close to the equilibrium values (i.e., alloy/slag mass ratio is 1.16 vs 1.17 for equilibrium). Observed Cr recovery and product qualities in terms of residual chromite/spinel compositions from our routine experiments at 1300 °C after 2 h are close to those of equilibrium values as well (alloy/slag mass ratio being 1.4 vs 1.67 for equilibrium). These observations suggest that reaction kinetics are such that near equilibrium conditions are reached at 1200 °C after 30 min and they are very close at 1300 °C after 2 h.

**Table 2:** Phase Quantities (wt %) in Products Representing Experiments 2 and 7, and Those Predicted under Equilibrium Conditions

	950 °C		1200 °C	
	exp 2	equilibrium	exp 7	equilibrium
alloy	1.8	7.8	53.6	53.9
spinel	95.5	78.4	31.7	30.2
olivine	0.7		6.6	11.9
silicate-slag	2.0	13.7	8.1	4.0
alloy/slag by mass	0.02	0.08	1.16	1.17

### 3.2 Material Characteristics

Experimental products include residual chromite, alloy, slag, and residual petcoke. Slag is dominated by spinel but includes forsterite, monticellite, merwinite, and glassy compounds that are interstitial, occupying space between alloy, chromite, and other slag phases. This interstitial slag phase is interpreted to represent a trapped residual melt.

**Figure 2:**
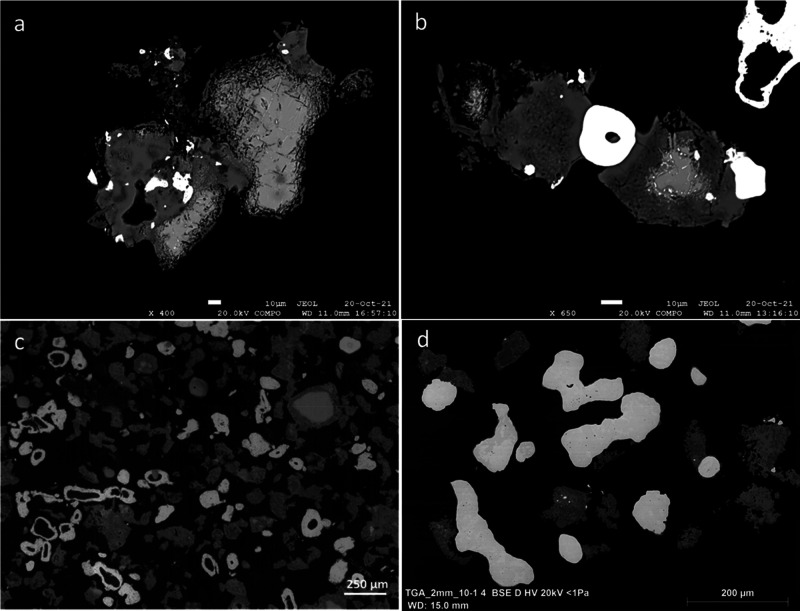
Backscatter electron (BSE) photomicrographs of reduced products (white: alloy; light gray: residual chromite; dark gray: interstitial slag and spinel; black: epoxy). (a) Residual chromite with dissolution pits developing along particle boundaries and crystallographic planes at 1200 °C after 30 min (exp#7 in Table 1). (b) Residual chromite particles with Cr-deficient (spinel) margins at 1300 °C after 1 min (exp#8). (c) Shrinking cores of chromite (light gray) and petcoke surrounded by atoll-like alloy formed after 15 min at 1300 °C (exp#9). A large residual chromite particle on the right is enveloped by residual Mg–Al spinel (dark gray) outlining the original chromite particle. With continued reduction all chromite and petcoke centers shrink and disappear. (d) Typical DRC product formed after 120 min of reduction at 1300 °C (exp#10).

Chromite particles in the 800 °C sample looked intact without any obvious dissolution features along grain boundaries. There is zoning, however, showing rims with increased Fe, developed in response to Mg–Fe^2+^ exchange reactions with the slag melt. Chromite grains in the 950 °C sample also display zoning features with thin Fe-rich rims. In addition, some chromite particles display corroded-looking outlines, probably indicating the beginning of incongruent dissolution of chromite. At 950 °C, the slag has occasional grains of forsterite (Mg_2_SiO_4_). The products formed between 1000 and 1300 °C are composed of variable quantities of chromite, forsterite, graphite (residual petcoke), interstitial slag, and alloy particles. Alloy particles in the 1000 and 1100 °C samples are small, measuring 1–5 μm across, forming thin slivers around carbon particles and occurring as discrete particles. At 1200 °C, chromite particles begin to display significant rimming and dissolution features along grain boundaries and crystallographic planes indicating the extent of its reaction with the melt (Figure 2a). With continued reaction, the rims become thicker at the expense of shrinking core of chromite (Figure 2b).

Overall, the dissolution of chromite is accompanied by the growth of ferrochrome at the expense of carbon particles. In essence, these features can be described as shrinking cores of chromite and reductant particles occurring in unison as the reactions proceed (Figure 2c). Following prolonged reaction at 1300 °C, the size and amount of M_7_C_3_-type carbide particles increase significantly while residual chromite becomes spinel (MgAl_2_O_4_) with minor Cr (Figure 2d). After 2 h of reaction at 1300 °C, the slag contains wadalite ((Ca,Mg)_6_Al_4_((Si,Al)O_4_)_3_O_4_Cl_3_), merwinite (Ca_1.5_Mg_0.5_SiO_4_), and monticellite (CaMgSiO_4_) in addition to spinel.

The carbon-deficient sample reduced at 1300 °C for 4 h, representing a situation where the molten slag is saturated with dissolved Cr, is made of spinel, forsterite, monticellite, and enstatite (MgSiO_3_) in addition to interstitial slag. The metallic phase is a M_7_C_3_-type alloy occurring in the form of discrete spherical particles. The sample with minor bentonite is composed of residual chromite, interstitial slag and alloy.

### 3.3 Evolution of Chromite Composition

**Figure 3:**
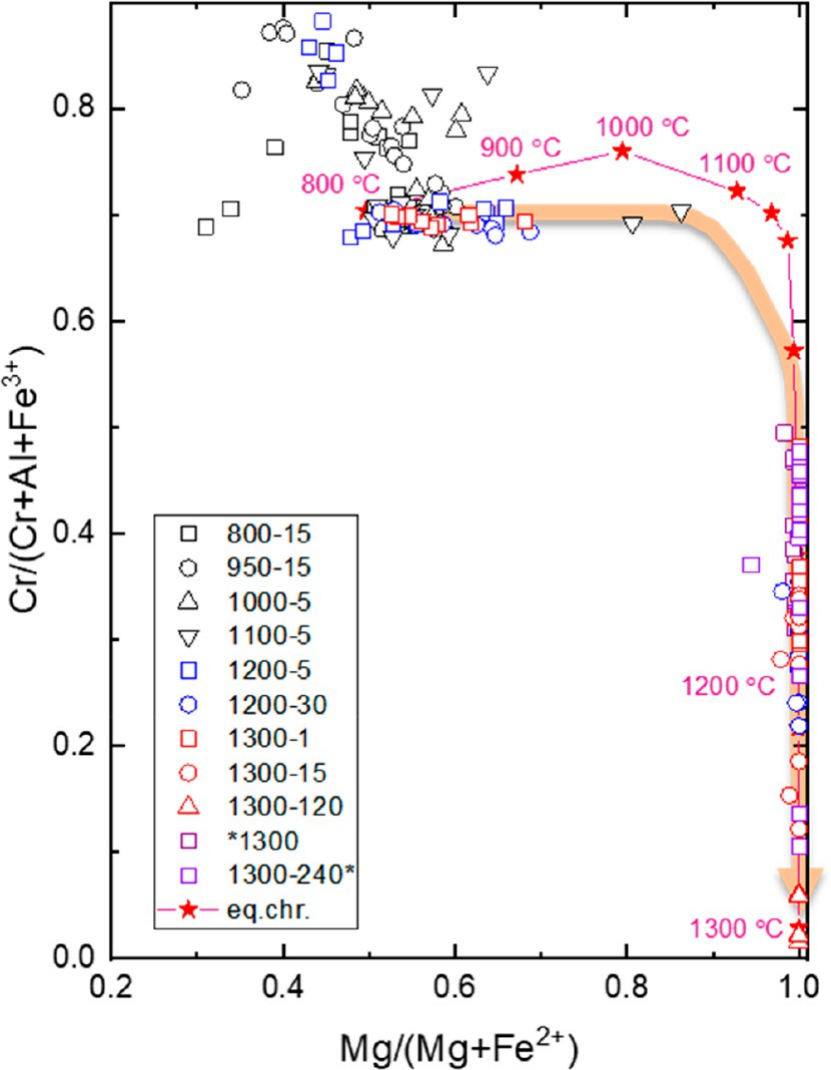
Changes in the composition of chromite with reduction in the temperature range of 800–1300 °C at various time intervals. Reduction path is highlighted by an orange arrow. Equilibrium compositions of chromite at 800–1300 °C calculated by FactSage 8.1 are shown by red star symbols.

Changes in the composition of chromite during reduction at temperatures from 800 to 1300 °C are shown in Figure 3. There is an overall increase in the proportion of Mg over the divalent cations from 800 to about 1100 °C and decrease of Cr over the total trivalent cations from about 1150 to 1300 °C, reflecting Fe and Cr losses from the spinel structure. Residual chromite in the products of 1200 °C and beginning of 1300 °C has two compositional groups. The first group of chromite displays similarities to those observed at lower temperatures with Cr/(Cr+Al+Fe^3+^) ratios that are around 0.7. These are essentially residual core regions of chromite particles that are not in equilibrium with the slag melt. The second group is characterized by Cr/(Cr+Al+Fe^3+^) ratios that are lower than 0.5 with corresponding Mg/(Mg+Fe^2+^) ratios of about 1. This signifies higher losses of Cr from chromite that is equilibrating with the slag melt through the diffusion of Cr from the core to outer regions where dissolution is occurring. The reduction path is dominated by increases in the Mg/(Mg+Fe^2+^) ratio during the early stages of reduction until about 1100 °C where 10% Fe^2+^ remaining in the tetrahedral sites. At this point, the reduction path makes a sharp turn, indicating the onset of Cr reduction with continual loss of Cr. All Fe^2+^ is reduced at about 1150 °C corresponding to 50% Cr remaining in the crystal structure. Following that, Cr reduction dominates until nearly all Cr is dissolved and reduced, with the resultant composition nearing MgAl_2_O_4_. Chromite compositions evolve from Mg_0.5–1.2_Al_0.5–1.4_Cr_0.6–1.3_O_4_ to Mg_1.0–1.1_Al_1.3–1.6_Cr_0.2–0.7_O_4_ and to Mg_1.0–1.2_Al_1.7–1.9_Cr_0.0–0.1_O_4_ with continued reactions after 1, 15, and 120 min at 1300 °C. This overlap in continuity of the chromite compositions signifies the shrinking core concept of chromite dissolution. Except for slight increases in the Cr/(Cr+Al+Fe^3+^) ratio from 800 to 1000 °C, which is resulting from reduction of Fe^3+^ in this temperature range, the changes in chromite composition are consistent with the predicted equilibrium compositions. This implies that the equilibrium is reached or very close after several hours of reaction at 1300 °C and above. There is minor to trace amounts of Cr remaining in the core regions of spinel at 1300 °C. These range from about 11 to 31 wt % Cr_2_O_3_ after 15 min of reduction to 1.39–5.82 wt % after 120 min. This is supported by the equilibrium compositions, implying that the residual Cr in spinel is not kinetically controlled, which limits Cr recoveries to 99.7 wt % at 1300 °C. Residual chromite in the sample with minor bentonite reduced at 1300 °C has a narrow compositional range (MgAl_1.2_Cr_0.8_O_4_).

### 3.4 Ferrochrome Composition

**Figure 4:**
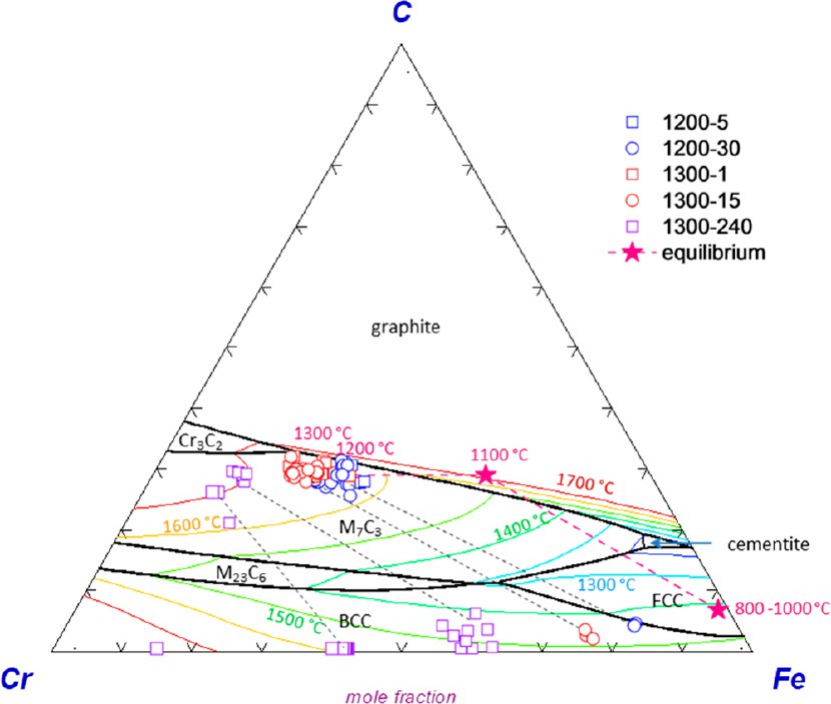
Alloy compositions (mole fraction) determined by electron probe microanalysis projected onto the Cr–Fe–C phase diagram. M_7_C_3_ and their coexisting austenitic alloys are connected by black dash lines. Equilibrium compositions of alloys forming at 800–1300 °C are those that were computed from the same feed composition used in experiments (star symbols for 1200 and 1300 °C not visible behind the experimental data points). Phase diagram is liquid projection at 1 atm computed by FactSage 8.1. Isotherms are drawn at 100 °C intervals.

Alloy compositions forming from 1200 to 1300 °C are shown in the Cr–Fe–C phase diagram (Figure 4). There is an overall enrichment in the Cr contents of M_7_C_3_ type carbide from 1200 to 1300 °C. At 1200 °C, alloy compositions evolve from Cr_4.1_Fe_2.9_C_3_–Cr_4.8_Fe_2.2_C_3_ after 5 min to Cr_4.4_Fe_2.6_C_3_–Cr_5_Fe_2_C_3_ after 30 min of reaction. Similarly, alloys evolve to higher Cr compositions with time and coexist with austenitic Fe with increased Cr content as the temperature increases to 1300 °C. After 4 h of continued reaction at 1300 °C, the alloys reach Cr_5.9_Fe_1.1_C_3_–Cr_6.5_Fe_0.5_C_3_. Coexisting austenitic alloys have compositions at Fe_0.6_Cr_0.4_-Fe_0.4_Cr_0.6_ with C concentrations reaching 1.4 wt % (Figure 4). The alloy compositions are in line with the predicted equilibrium M_7_C_3_ compositions representing 1200 and 1300 °C (Figure 4). For instance, the alloy compositions resulting from reduction at 1300 °C (i.e., Cr_4.7_Fe_2.3_C_3_) is close to the equilibrium composition of Cr_4.9_Fe_2.1_C_3_, suggesting near-equilibrium conditions after 2 h of reduction at 1300 °C.

The Cr/Fe mass ratios of the ferrochrome particles formed after 15 min of reaction at 1300 °C (exp no. 9 in Table 1) are in the 2.1–3.0 range which covers the Cr/Fe ratio of the ore in the feed (i.e., 2.1–2.2), suggesting that the alloy composition reaches its equilibrium value before the bulk of Cr in chromite is reduced. This is also the case for the ring-shaped alloy particles formed after 30 min of reaction at 1200 °C. They have compositions of Cr_4.3–4.6_Fe_2.4–2.7_C_3_ nearing equilibrium value of Cr_4.7_Fe_2.3_C_3_, suggesting that the alloy composition evolves fast with the reduction and transport of Cr species.

### 3.5 Interstitial Slag Composition

**Figure 5:**
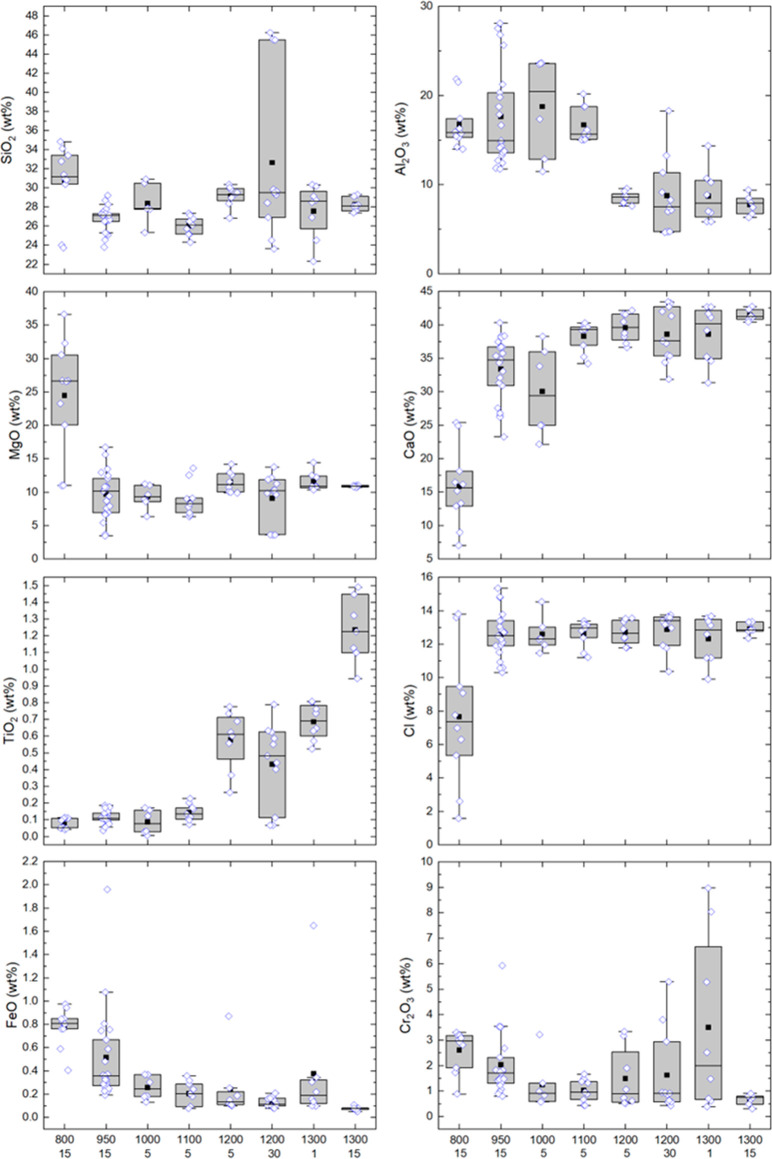
Interstitial slag compositions at temperatures of 800–1300 °C based on electron probe microanalyses (blue symbols). Box charts cover the data that fall in the 25th and 75th percentiles with the median represented by the center line and the whiskers outlining the spread within 1.5 times the interquartile ranges. Numbers below the temperatures are dwell times in minutes.

Compositions of the interstitial slag that formed at various temperatures and reaction times are summarized in Figure 5 and shown in Table S2. Except for CaO and Cl, the interstitial slag formed at 800 °C is compositionally similar to that of clinochlore^15^ (SI Table S1), suggesting congruent dissolution of clinochlore in forming the slag at this temperature. In terms of overall concentrations, interstitial slags formed between 1200 and 1300 °C at 15 min can be grouped together with the average values of 28.16 ± 2.02 wt % SiO_2_, 8.85 ± 2.60 wt % Al_2_O_3_, 39.82 ± 3.23 wt % CaO, 11.29 ± 1.26 wt % MgO, 0.75 ± 0.31 wt % TiO_2_ and 12.64 ± 0.99 wt % Cl representing 31 compositions. The interstitial slag formed at 1300 °C after 15 min of reaction displays a much narrower compositional range. These compositions are similar in terms of CaO, Al_2_O_3_, MgO and FeO concentrations to the typical blast furnace slag.^16^ Silica concentrations are lower than that of the blast furnace slag, which is in the 27 to 38 wt % range. Interstitial slag formed at 950 °C and above have a rather narrow range of Cl concentrations in the 12 to 14 wt % range (Figure 5), suggesting mixing of CaCl_2_ liquid with the slag melt. This is supported by the solubility data of CaO in molten CaCl_2_ which is about 33 mol % at 700–835 °C,^17^ and 20 mol % up to 840 °C and 29 mol % at 1300 °C as per FactSage predictions. Inferred from the similarity of the average composition of the 1300 °C slag to wadalite in terms of CaO and Cl concentrations (Table 3), chlorine is eventually fixed in the slag as wadalite upon cooling.

**Table 3:** Composition of the Various Slags Based on Electron Probe Microanalyses (wt%)^a^

	Slag A	Slag B	Slag C	Slag D	Slag E
n	10	7	3	23	18
SiO_2_	30.64 ± 3.86	28.26 ± 0.72	28.35 ± 0.33	36.33 ± 0.32	26.51 ± 0.45
TiO_2_	0.07 ± 0.03	1.24 ± 0.20	0.08 ± 0.05	1.09 ± 0.03	0.53 ± 0.03
Al_2_O_3_	16.76 ± 2.76	7.76 ± 1.04	12.56 ± 0.23	18.84 ± 0.81	2.15 ± 0.05
Cr_2_O_3_	2.60 ± 0.81	0.67 ± 0.21	0.12 ± 0.07	3.46 ± 0.30	0.91 ± 0.20
FeO	0.77 ± 0.17	0.08 ± 0.02	0.04 ± 0.05	0.06 ± 0.03	0.22 ± 0.13
CaO	15.84 ± 5.96	41.43 ± 0.81	47.34 ± 0.23	34.28 ± 0.61	46.96 ± 0.66
MgO	24.47 ± 8.45	10.89 ± 0.12	9.15 ± 0.13	1.58 ± 0.26	5.30 ± 0.16
Cl	7.65 ± 4.06	12.91 ± 0.34	4.06 ± 0.08	4.04 ± 0.18	22.09 ± 0.35

	Slag F	Slag G	wadalite	wadalite^1^	wadalite^2^
*n*	21	9	24		11
SiO_2_	29.12 ± 0.40	45.74 ± 0.43	12.2–19.1	15.35	15.66
TiO_2_	0.53 ± 0.02	0.08 ± 0.04	0–0.4		0.72
Al_2_O_3_	2.15 ± 0.04	4.72 ± 0.06	24.3–33.4	27.76	23.06
Cr_2_O_3_	1.28 ± 0.17	0.90 ± 0.09	0.2–1.2		
FeO	0.03 ± 0.01	0.19 ± 0.04	0–0.1	4.22	7.25
CaO	44.36 ± 0.30	35.08 ± 0.61	35.3–43.9	42.13	41.55
MgO	8.57 ± 0.36	3.62 ± 0.06	2.7–10.5	1.18	1.68
Cl	18.47 ± 0.47	13.47 ± 0.25	4.8–10.8	11.96	9.79

a *n*: number of analyses; standard deviations are based on 3σ; Slag A: 800 °C, 15 min (exp#1 in Table 1); Slag B: 1300 °C, 15 min (exp#9); Slag C: 1300 °C, 120 min (exp#10); Slag D: 1300 °C, 180 min (exp#12 containing minor bentonite); Slag E and F: 1300 °C, 240 min (exp#11) coexisting with large FeCr grains and stringers (SI-Figure 6); Slag G: 1200 °C, 30 min (exp#7) representing Type 4 Cr species; wadalite from exp#9 at 1300 °C, 120 min; wadalite^1^ (Tsukimura et al.);^18^ wadalite^2^ (Mihajlovic et al.).^19^

The interstitial slag representing the feed that contained minor bentonite displays differences from those of 1300 °C in terms of its SiO_2_ and Al_2_O_3_ concentrations (SI Table S2), reflecting the addition of bentonite to the feed. Interstitial slags originating from the carbon-deficient feed after 4 h of reaction at 1300 °C have the highest CaO and Cl, and lowest Al_2_O_3_ concentrations among the slag samples (Table 3). Wide variability of interstitial slag compositions observed in some samples (e.g., 1200-30) reflects the presence of local pools of melt trapped rather than equilibrium compositions.

On the CaO-MgO-SiO_2_ ternary phase diagram, interstitial slag compositions representing 800 °C plot in the olivine field forming a trend from forsterite (Mg_2_SiO_4_) toward melilite (Ca_2_MgSi_2_O_7_) (Figure 6). This trend follows the down-temperature liquidus surface, indicating that the slag melt is saturated with respect to forsterite. Since there is no olivine observed in this sample, this is likely to be indicative of the nucleation and formation of nanosized forsterite at this temperature. Mass balance calculations suggest the nucleation of nanosized forsterite between 19 to 64 wt %. Interstitial slag compositions of 950 and 1000 °C experiments form two clusters: one in the melilite field and another in the merwinite (Ca_3_MgSi_2_O_8_) and Ca-olivine (Ca_2_SiO_4_) fields straddling the forsterite-Ca-olivine compositions. Interstitial slags that formed above 1100 °C cluster along the joins of MgO with Ca-olivine and merwinite fields with the exception of a few representing 1200 °C after 30 min.

**Figure 6:**
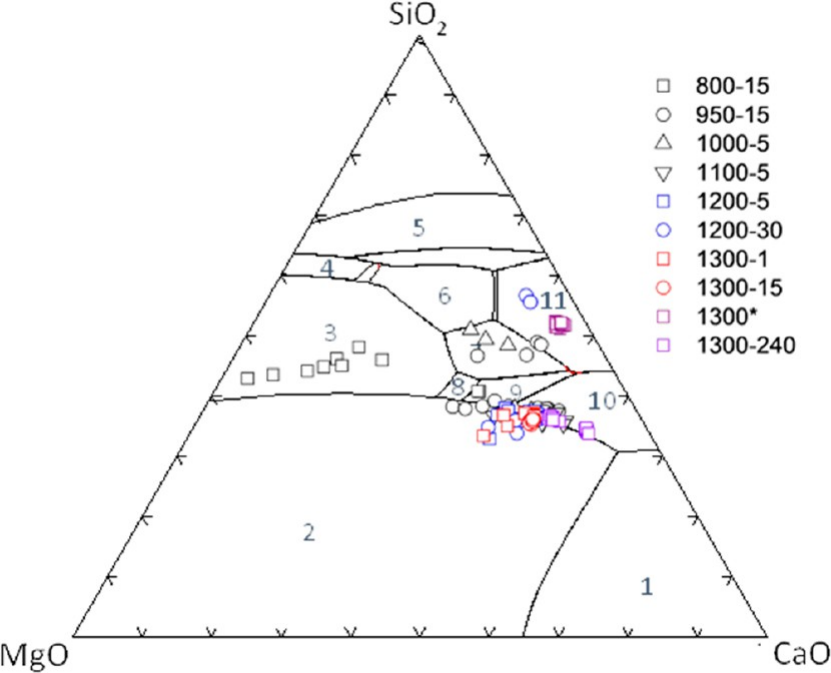
CaO, MgO, and SiO_2_ mass fractions of slags formed at 800–1300 °C as shown on the CaO-MgO-SiO_2_ ternary phase diagram. 1: monoxide CaO; 2: monoxide MgO; 3: olivine; 4: protopyroxene; 5: quartz; 6: clinopyroxene; 7: melilite (Ca_2_MgSi_2_O_7_); 8: monticellite (MgCaSiO_4_); 9: merwinite (Ca_3_MgSi_2_O_8_); 10: Ca-olivine (Ca_2_SiO_4_); 11: wollastonite (CaSiO_3_). Phase boundaries were calculated using FactSage 8.1.

Interstitial slags have highly variable chromium concentrations from 0.3 to 8.97 wt %. Concentrations reaching 5.9 wt % Cr_2_O_3_ in the 800 and 950 °C interstitial slags are likely to be originating from the dissolution of clinochlore containing 3.62 wt % Cr_2_O_3_ (SI Table S1). Increased levels of Cr concentrations in the interstitial slag coincide with the occurrence of chromite particles having rimmed margins beginning at 1200 °C (Figure 2). Highly variable Cr_2_O_3_ concentrations in interstitial slag at the beginning of 1300 °C are stabilized at 0.3 to 0.9 wt % after 15 min of reduction (Figure 5). This supports the earlier assertion made on the rapid equilibration of ferrochrome before the bulk of Cr in chromite is reduced. These Cr_2_O_3_ concentrations are comparable to the solubility values that are in the 0.3 to 1.4 wt % range for melts saturated with chromite at 1200–1630 °C.^20−22^ Reported chromium solubilities in slags and basaltic magmas are highly variable from 0.2 to 5.8 wt % Cr_2_O_3_ depending on the melt composition, temperature and oxygen partial pressure.^20−25^ Nell (2004)^26^ reported solubility values ranging from 1 to 6 wt % Cr_2_O_3_ at 1550 °C. Chromium solubility appears to be greater at lower oxygen partial pressures.^20,21,23,25^ In addition, Cr solubility appears to increase with temperature.^20−22^ The range of Cr_2_O_3_ concentrations in our interstitial slags is broadly comparable to these solubility values. The Cr_2_O_3_ concentrations of 0.3–0.9 wt % in the 1300 °C interstitial slag would be well below the solubility limits of slag characteristic of the DRC process, because they are representative of melt compositions when chromite dissolution and Cr reduction reactions are occurring in unison. Furthermore, Huang et al.^27^ reported that large concentrations of Cr can be transported in Cl-rich melts under reducing conditions, implying that Cr solubility is also dependent on the Cl concentrations. These suggest that accelerated reduction of Cr and formation of M_7_C_3_ alloys are limiting the buildup of Cr concentrations in molten slag. The Cr_2_O_3_ concentrations of about 15 wt % in interstitial slags that formed at 1300 °C after 4 h of reaction with substoichiometric carbon would represent a scale of high Cr solubility in molten slag.

Interstitial slag compositions that resulted from the feed amended with bentonite are also comparable (Slag D in Table 3). An interesting observation is the presence of 3.46 ± 0.30 wt % Cr_2_O_3_ in this interstitial slag. It is possible that this slag is in equilibrium, with the residual Cr-spinel occurring as dispersed particles embedded in the slag. This is in part due to the refractory nature and narrow compositional range of the residual Cr-spinel (MgAl_1.0–1.3_Cr_0.6–1.0_O_4_). In this case, the Cr_2_O_3_ concentration of the interstitial slag can be representative of the solubility limit of this melt.

### 3.6 Speciation of Cr

**Figure 7:**
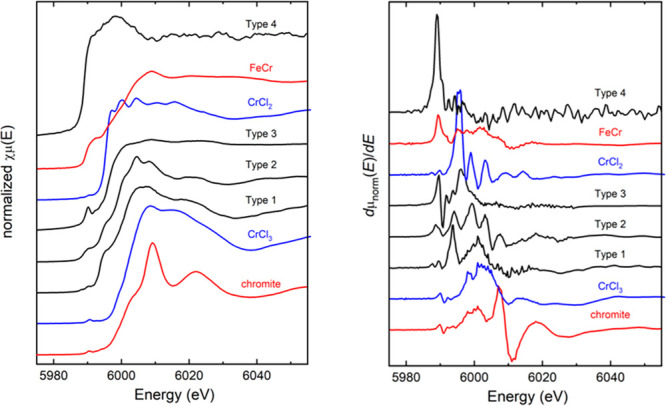
Cr *K*-edge micro-XANES spectra (left: normalized; right: derivative) collected from Type 1–4, chromite, FeCr alloy, and Cr-chloride references. With increased reduction, the edges of the slags shift from Cr^3+^ as in chromite to Cr^0^ as in the FeCr alloy. The pre-edge 1*s* → 3*d* is at 5989.5 eV and the 1*s* → 4*s* transition at 5992–5994 eV. Vertical scales of the spectra and their derivatives are identical.

Distribution of Cr species was delineated by backscatter electron imaging, electron probe microanalyses, wavelength dispersive X-ray mapping and micro-XRF mapping. Based on more than 350 micro-XANES spectra collected from interstitial slag, residual chromite rims and olivine, four distinct types of reduced Cr species can be identified (SI Table 4). Shifts in the absorption edges span an energy range reflective of changes in the oxidation states from 3+ to 0 (Figure 7).

**Table 4:** Local Structural Parameters of the Slag Determined from Fitting of Cr *K*-Edge Micro-EXAFS Spectrum^a^

	CN	*R*	σ^2^	E0	rf	χ^2^
Cr–O	5^b^	2.01 ± 0.01	0.0072 ± 0.0010	4.2 ± 0.9	0.0029	33
Cr–Cl	1^b^	2.68 ± 0.04	0.0113 ± 0.0068			

a CN: coordination number; R interatomic distance (Å); σ^2^: Debye–Waller parameter (Å^2^); E0: energy offset (eV); rf (r-factor) and chi-sq (chi square) as the goodness-of-fit parameters. Fit performed in R-space with R = 1–3 Å, k = 3–11 Å^–1^ and amplitude-reduction factor (S_0_^2^) constrained to 0.9;

b fixed; Number of independent points/number of variable value is 8.69/5.

Type 1 is characterized by spectral features at 5989.5, 5994, and 6001 eV (Figure 7) which are similar to those of Cr^2+^ species in borosilicate glass and lunar olivine,^28^ silicate glasses^29^ (SI Figure S1) and Cr^2+^ standard.^30^ The derivative peaks at ∼5989 and 5994 eV below the main peak at around 6001 eV are assigned to 1*s* → 3*d* and 1*s* → 4*s* transitions as per Waychunas et al. (1983)^31^ and Sutton et al. (1993).^28^ Type 2 is characterized by the pre-edge feature at 5989.7 eV and absorption edges at 5994, 5999, and 6003 eV (Figure 7). Similar to the Type 1 Cr species, the edge at 5994 eV is representing Cr^2+^ coordinated to oxygen whereas the edge at 6003 eV is an indication of the presence of Cr^3+^. Type 3 has its main absorption edge at 5995.5 eV, slightly at a higher energy position than those of Type 1 and 2 (Figure 7). It aligns with that of CrCl_2_ indicating coordination of the absorbing atom with chlorine. Although minor, Type 3 has another edge at 5994 eV which lines up with oxygen-coordinated Cr^2+^ edges of Types 1 and 2 such as glass and olivine. These observations suggest that Cr^2+^ of Type 3 is coordinated to not only Cl but also O. Furthermore, XANES spectrum of Type 3 species resembles several organochromium(II) complexes coordinated to Cl, P, O, and C, and formed after rapid methylation of Cr^3+^ and reacting with trimethylaluminum in toluene.^32^ With its absorption edge at 5989 eV, similar to those of M_7_C_3_-type ferrochrome, Cr_3_C_2_ and Cr metal, Type 4 represents the most reduced Cr species (Figure 7). The XANES spectral features shown by four types of Cr species are likely to be influenced by not only the oxidation state and coordination chemistry^31,33^ of Cr but also from the differences in electronegativity of ligands like O^2–^ and Cl^–^.^34,35^

**Figure 8:**
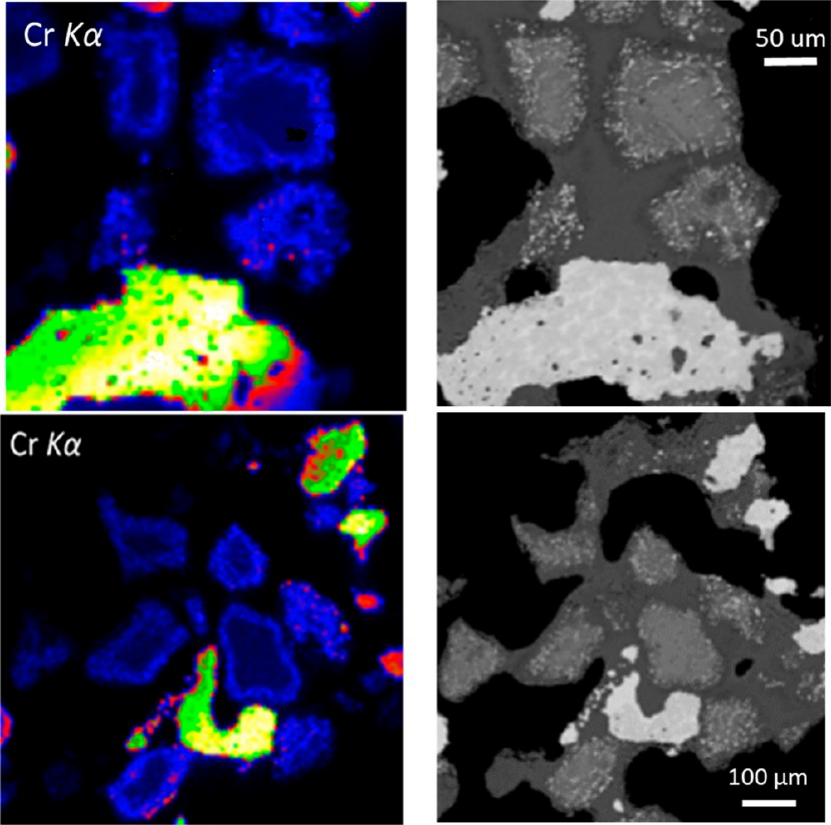
Synchrotron micro-X-ray fluorescence map (sXRF) images showing the distribution of Cr near the sample surfaces (left) with the corresponding BSE images shown on the right. Similarity of the sXRF maps to the BSE images confirm that the signals from deeper parts of the sample were effectively filtered out by the confocal optics. On the sXRF images, residual chromite particles appear as dark bluish black whereas the rims with disseminated FeCr alloy particles are bright blue. FeCr alloy occurs as green, yellow, and red particles, reflecting different Cr counts from the Cr- and Fe-rich zones. Type 1 Cr species is uniformly distributed in the interstitial slag areas shown as dark gray areas in BSE photomicrographs. The slag has low Cr contents of about 3.5 wt % as Cr_2_O_3_ and appears as a black background on the sXRF maps.

Type 1 represents molten slag that formed at 1300 °C after 3 h of reduction (Figure 8; exp no. 12 in Table 1). Type 1 species is also inferred to be present in products formed at 1200 °C after 30 min (SI Figure 3), immediately at 1300 °C and after 4 h at 1300 °C (Table 1, exp nos. 8 and 11). Using the approach of Berry and O’Neill^29^ in integrating the area of 1s → 4s peak and with the assumption that Cr^2+^ coordination environments are the same, the proportion of Cr^2+^ in Type 1 slag can be estimated to be 90% of total Cr. The proportion of Cr^2+^ could be higher than this or it is entirely Cr^2+^ depending upon the compositional influence and partial oxygen pressure. Oxygen partial pressure under which the slag formed is estimated to be close to 1 × 10^–16^ atm, representing the C–CO equilibrium which would promote the highest proportion of Cr^2+^ in the slag. This finding agrees with the FactSage-predicted equilibrium value of 97% in the slag melt at 1300 °C. Occurrence of Cr^2+^ in slag melt is supported by earlier experimental studies.^20,21,23,24,36^ These studies indicate that the speciation of Cr in slag and magmas is controlled by temperature, partial pressure of oxygen, and composition. For instance, the proportion of Cr^2+^ over the total Cr is 33% in a melt containing 20 wt % MgO with equal mass proportion of CaO and SiO_2_ at 1600 °C and oxygen partial pressure of 2.73 × 10^–10^ atm whereas it reaches 60% with an increase of the Al_2_O_3_ content of the melt to 23 wt %. Studies of Hanson and Jones (1998)^21^ on the partitioning behavior of Cr in basaltic magmas at an oxygen partial pressure of 10^–11^ atm indicate that the proportion of Cr^2+^ is 61% at 1200 °C and 76% in the absence of Fe at 1320 °C. Based on Roeder and Reynolds (1991)^20^ experimental results, the proportion of Cr^2+^ in basaltic magmas saturated with chromite can be inferred to reach 94% at 1300 °C and 10^–13^ atm. Overall, the proportion of Cr^2+^ increases with a decrease in the partial pressure of oxygen.^20,23^ If we extrapolate the findings of Hanson and Jones (1998)^21^ to lower oxygen partial pressures that are more relevant to the DRC process, the proportion of Cr^2+^ becomes 96–99% at 10^–16^ atm. This is indicative of a near complete reduction of Cr species in liquid upon reaching equilibrium.

**Figure 9:**
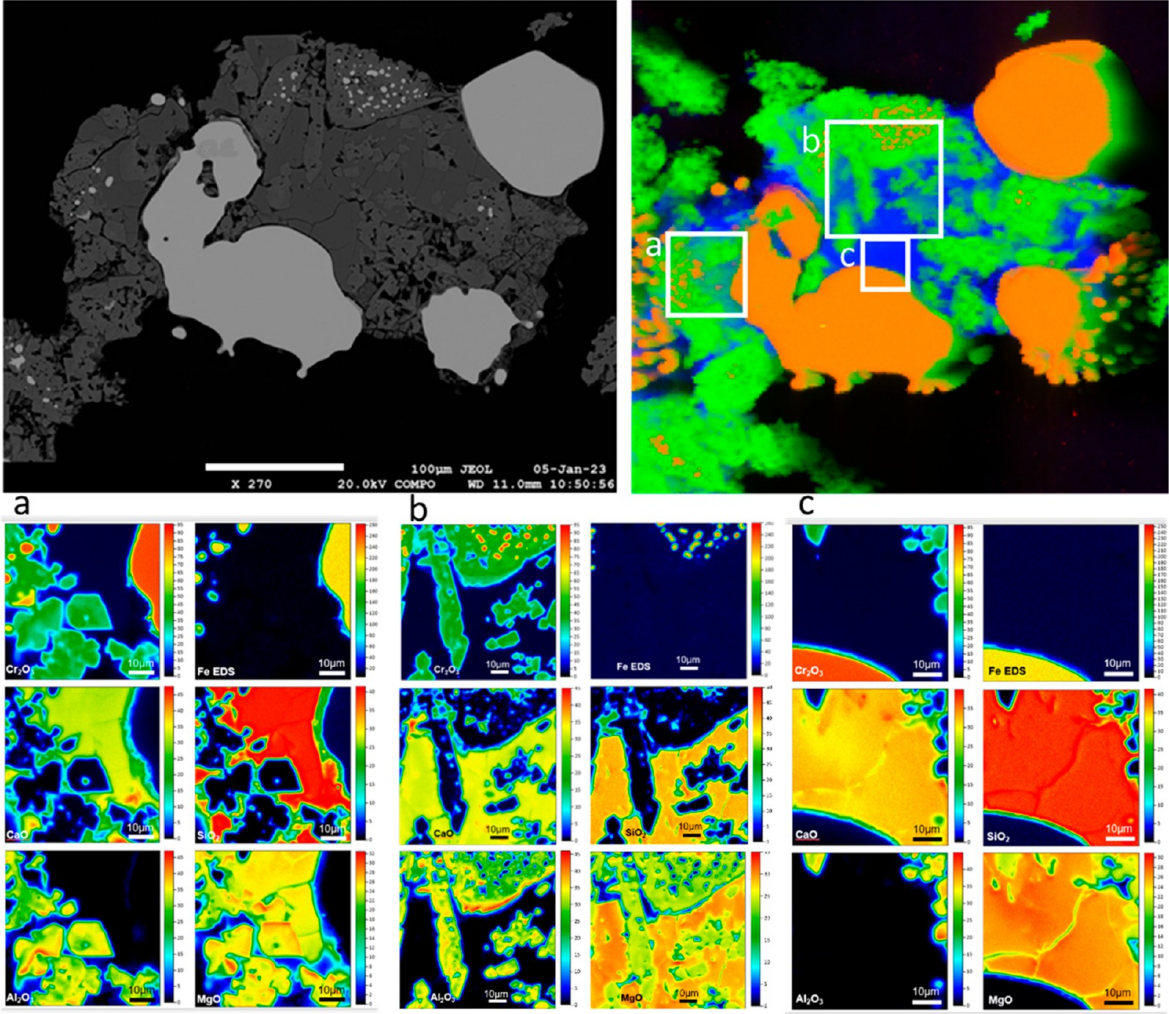
BSE photomicrograph (upper left) and sXRF map (upper right) showing monticellite (dark gray; blue), residual chromite (lighter gray; green), and FeCr (white; orange). Quantitative wavelength-dispersive X-ray maps (qWDS) (bottom panels) show distribution of SiO_2_, Al_2_O_3_, Cr_2_O_3_, MgO, CaO, and Fe in the areas outlined on the upper right sXRF map (a–c). Micro-XANES spectra indicate that monticellite has Type 2 species. Chromite rims are either Type 3 or a mixture of Type 3 with minor chr.

Type 2 is typically observed in monticellite formed at 1300 °C after 4 h of reaction (Figure 9). Monticellite occurs as a groundmass to chromite and alloy particles, suggesting that it formed from the molten slag during cooling. Its composition is Mg_1.1_Ca_0.9_SiO_4_ based on electron probe microanalyses and quantitative wavelength dispersive X-ray maps (Figure 9). It contains 1.94 ± 0.52 wt % Cr as Cr_2_O_3_. Type 2 species is also inferred to be present in various slags formed at 1200 and 1300 °C (SI Table S3; SI Figures S2–S12).

**Figure 10:**
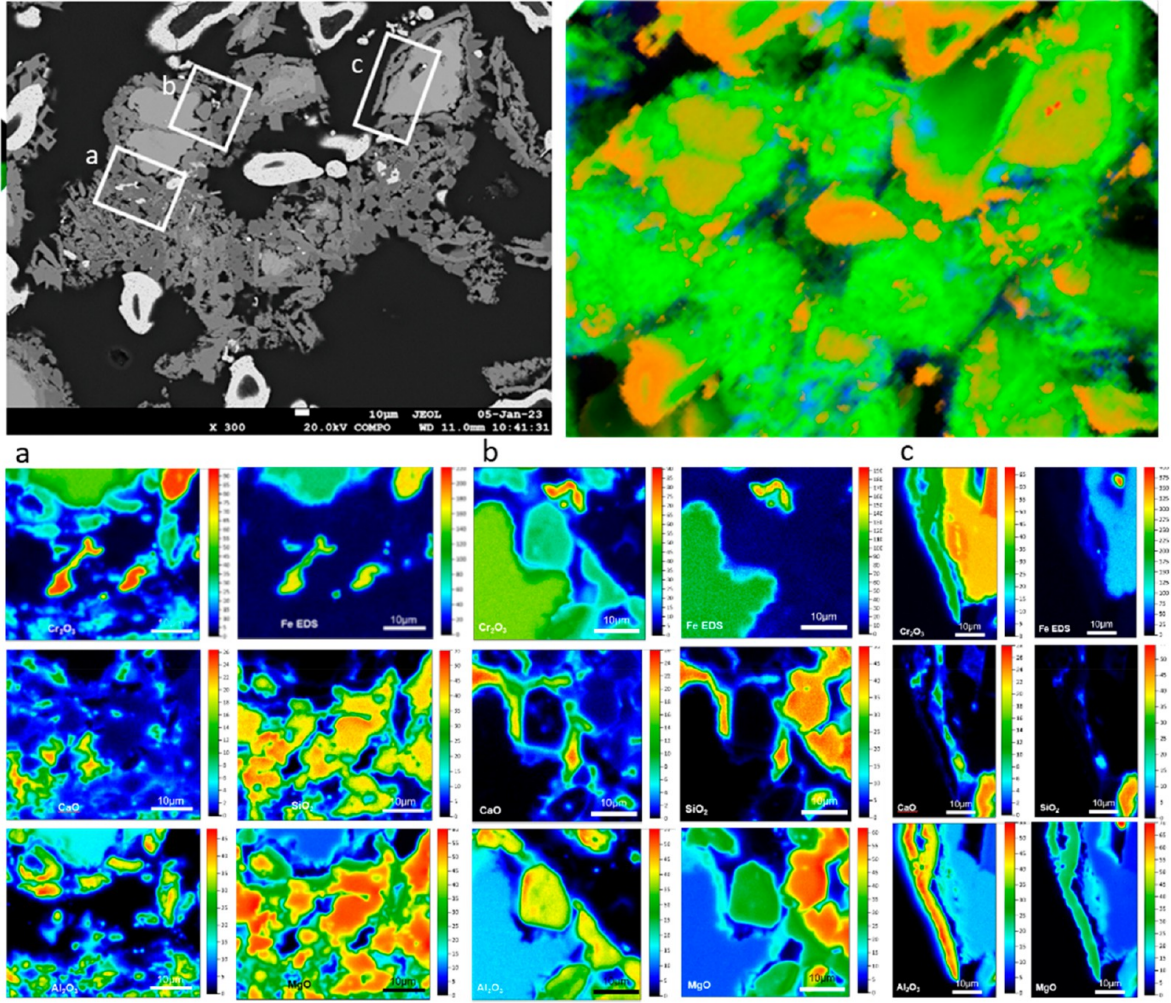
BSE and sXRF maps showing chromite rim (dark gray; green) and slag areas (dark gray; green) dominated by Type 3 Cr species in a sample representing 1200 °C after 15 min reaction. Details of the areas marked by rectangles (a, b, and c) are shown by qWDS maps on the bottom panels. Light orange and dark orange areas on the sXRF maps depict residual chromite and alloy particles, respectively. Chromite rims have Type 3 species with smaller proportions of Type 4, Type 2 and chromite (Cr^3+^).

Type 3 Cr species is typically observed in chromite rims (Figures 9 and 10) with a composition of MgCr_0.6–0.7_Al_1.2–1.3_Mg_0.1_O_4_ in run products formed after 4 h of reaction at 1300 °C representing carbon deficiency (Table 1). Its association with chromite rims probably reflects the presence of pockets of slag melt in the porous residual spinel formed during incongruent dissolution of chromite. In addition, Type 3 is observed in a Ca–Mg aluminosilicate slag with Cl (SI Figures S13 and S14) that formed at 1100 °C after 5 min of reaction (Table 1). This slag having about 1 wt % Cr_2_O_3_ is compositionally similar to wadalite; however, it has slightly higher SiO_2_ and lower Al_2_O_3_ contents (Table 3). Another Ca–Mg aluminosilicate with Cl slag formed at 1300 °C after 2 h has 15% Type 3 species in addition to zerovalent Cr species represented by 55% FeCr and 30% Type 4 (SI Figure S15). It appears that Type 3 is the most abundant Cr species present in the products representing the experimental range from 1100 to 1300 °C (SI Figurse S2–S15). The presence of Type 3 species in both the chromite rims and interstitial slag suggests that the chromite rims equilibrated with a slag melt containing reduced Cr species.

**Figure 11:**
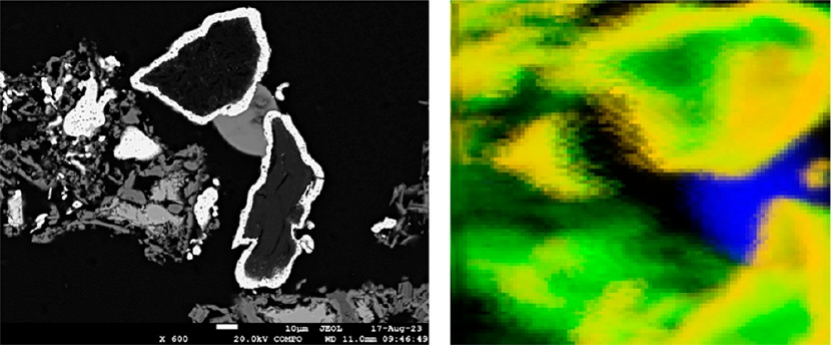
Backscattered electron image (left) and synchrotron X-ray fluorescence map (right) showing the interstitial slag possessing Type 4 Cr species (Slag G on Table 3). It is between two ring-shaped alloy particles shown as gray on the left image and blue on the right. Alloy composition is Cr_4.3–4.6_Fe_2.4–2.7_C_3_.

Type 4 observed in a Ca–Si–Cl slag that formed at 1200 °C after 30 min of reduction (Figure 11) is considered Cr^0^. This Cr species is also inferred to be present in a variety of interstitial slags (SI Table S3; SI Figure S15) indicating the reduction of Cr in molten slag soon after its dissolution from chromite beginning at 1200 °C.

Least squares fitting of the XANES spectra collected from interstitial slag and chromite rims indicate that they are in fact mixtures of Cr^3+^, Cr^2+^, and Cr^0^ species represented by chromite, interstitial slags, and alloy (SI Table S3; SI Figure S16).

### 3.7 Local Structure of Cr in Interstitial Slag

**Figure 12:**
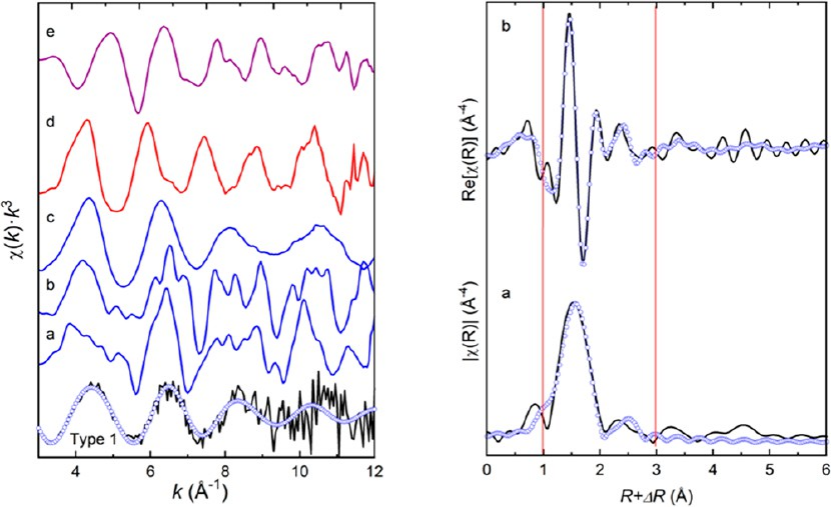
Chromium *K*-edge micro-EXAFS spectra of the Type 1 slag and reference compounds of chromite (a), Cr_2_O_3_ (b), CrCl_3_ (c), CrCl_2_ (d), and Cr_3_C_2_ alloy (e) (left panel). Measured spectrum shown in black, and the fit in *k*-space shown in blue circles. Fourier transform (a) and real part (b) of the experimental spectrum shown by solid black lines and their fits in blue circles. Fitting was performed in *R*-space within 1–3 Å, as marked by red lines on the right panel.

Chromium *K*-edge micro-EXAFS spectra collected from the Type 1 slag and various relevant reference materials are shown in Figure 12. Type 1 spectrum represents an average of 10 scans collected from various parts of the interstitial slag areas forming the interstices of residual chromite and alloy particles, as discussed earlier. Although the spectrum is noisy, oscillations are resolvable for the *k*-range of up to about 11 Å^–1^ for determining the local structure. The spectrum is broadly different from the spectra of the reference species of Cr^3+^, Cr^2+^, and Cr^0^ (Figure 12). The spectrum was simulated within the k range from 3 to 11 Å^–1^ by various combinations of first shell paths of Cr–O and Cr–Cl derived from a range of compounds like, chromite, Cr_2_O_3_, CrO, Cr(C_7_O_2_)_3_, CrCl_2_, and CrCl_3_. One of the best fits was obtained with the use of Cr–O paths of Cr(C_7_O_2_)_3_ where Cr is 2+.^37^ Cr–O radial distances were 1.95 and 2.11 Å with corresponding coordination numbers of 2.8 and 2.2 totaling 5 oxygen atoms. The second best-fit was obtained with the paths derived from a CrO compound^37^ and CrCl_2_ structure^38^ where Cr is divalent. Among the two, the fit represented by split 2 shells of oxygen and chlorine atoms was better in terms of fit quality and parameters. This fit involved 5 oxygen and 1 chlorine atoms at radial distances of 2.01 and 2.68 Å, respectively (Table 4). Addition of Cr–Cr paths was not successful in both simulations, corroborating the absence of higher shell neighbor atoms from the Fourier transform of the spectrum (Figure 12).

In the absence of second shell atoms, it is concluded that Cr(O,Cl) occurs in the form of monomers, probably afforded by the dilute nature of Cr in the molten slag. Electron microprobe analyses indicate that CrO content of this interstitial slag is relatively uniform at 3.10 ± 0.27 wt %. This slag is dominated by SiO_2_, CaO and Al_2_O_3_ with minor Cl and MgO (Table 3). In the case of Type 1 Cr species, it is contemplated that Cr is transported in molten slag as species that are 6-fold coordinated to five oxygen atoms and one chlorine atom forming monomers.

Local structures of the other Cr species cannot be determined due to the poor quality of their micro-EXAFS spectra. It is possible that dissolved Cr^3+^ coordinates to Cl in molten slag (i.e., exchanges O with Cl) while it is reduced to Cr^2+^ similar to reduction of Cr from 3+ to 2+ and 1+ in olefins as envisaged by Bartlett et al. (2016).^32^

### 3.8 Mechanism of the Process

Our new findings on the presence of reduced Cr species in slag indicate that our working hypothesis on the CaCl_2_-assisted DRC process was incomplete. With the new knowledge, the mechanism of DRC can be described as (1) incongruent dissolution of chromite, (2) reduction of Cr in molten slag, (3) transport of Cr and Fe species in molten media, and (4) further reduction on carbon particles and growth as Cr–Fe alloys.

Dissolution of chromite in molten CaCl_2_ can be described by reaction 1 where the reducible cations are depicted as dissolved Cr_2_O_3_ (*l*) and FeO (*l*) species in melt:
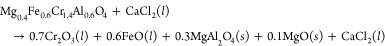
1

Incongruent dissolution of chromite begins at 800 °C with an increase in the Fe concentrations in the slag liquid. This is closely followed by the dissolution of Cr, leaving behind refractory spinel (MgAl_2_O_4_). Dissolution of chromite is controlled by the solubility limits of Cr and Fe in molten salt and slag liquid, requiring that the melt remain undersaturated with respect to Fe and Cr. It is estimated that the Cr_2_O_3_ concentrations of 0.3–0.9 wt % in interstitial slag are well below its solubility controlled by chromite and the low partial pressure of oxygen. At 1200 °C, Cr and Fe as dissolved species are transported in molten media through the porous spinel to the reduction sites on carbon particles. This kept the concentration of dissolved species below the saturation limit. Our results indicate that, after 2 h of reduction at 1200 °C, 92% of Fe and 59% of Cr are reduced. As the temperature reaches 1300 °C, all Fe species are reduced, and reactions involve only Cr reduction. At 1300 °C, about 95% Cr metallization is achieved after 2 h. Alloy composition changes from Cr_4.1_Fe_2.9_C_3_ to Cr_5_Fe_2_C_3_ reflecting increased Cr releases from shrinking chromite particles. Coexisting austenitic Fe also shows increases in the Cr content from Fe_0.9_Cr_0.1_ at 1200 °C to Fe_0.8_Cr_0.2_ at 1300 °C with C concentrations ranging from 0.8 to 1.4 wt %. Progress of reduction and metallization based on residual chromite/spinel and alloy compositions agree with our earlier estimates based on XANES spectroscopy performed on bulk products.^8^ These were 91.6% at 1200 °C and 100% at 1300 °C for the degrees of Fe metallization and 58.6% at 1200 °C, 94.2% at 1300 °C and 100% at 1400 °C for the degrees of Cr metallization.

Reduced Cr species occur in slag that formed as early as 1100 °C indicating that Cr was reduced to Cr^2+^ and Cr^0^ in the melt soon after its dissolution from chromite but before reaching solid carbon particles. The presence of Type 4 reduced Cr species in slag that formed after a prolonged reaction at 1300 °C following the depletion of solid carbon is noteworthy (SI Figures S4–S6). This slag occurs as a matrix to FeCr stringers that are devoid of carbon (Cr_3_Fe_2_) emanating from a Cr-rich M_7_C_3_-type alloy (Cr_6.5_Fe_0.5_C_3_), suggesting supersaturation of reduced Cr in molten slag. The FeCr stringers have 56.85 ± 1.33 wt % Cr, whereas the M_7_C_3_-type FeCr from which the stringers are radiating has much higher Cr content at 81.74 ± 0.56 wt %. Among the other phases nearby, residual chromite has about 34 wt % Cr_2_O_3_ (MgAl_1.2_Cr_0.7_Mg_0.1_O_4_) and monticellite forming another matrix component contains 1.64 ± 0.12 wt % Cr_2_O_3_ (SI Figures S4–S6).

As demonstrated by micro-XANES and EXAFS experiments, the interstitial slag at 1300 °C is dominated by divalent and more reduced species of Cr and is 6-fold coordinated to oxygen and chlorine. Absence of higher shell contributions to the EXAFS spectra points to the occurrence of Cr(O,Cl) as a monomeric species in molten slag during their transport to reduction sites. The presence of Cr^2+^ (Type 3 species) in the rims of chromite as well as in interstitial slag suggests that immediately after its dissolution from chromite, Cr_2_O_3_ is reduced to CrO due to low oxygen partial pressure. This finding refutes our original contention of higher oxygen partial pressures of about 10^–10^ atm near chromite particles.^9^

Reduction of CrO to Cr metal (reaction 2) is thermodynamically feasible at a lower temperature (i.e., >1075 °C) than it is for Cr_2_O_3_ which is at >1145 °C (reaction 3).
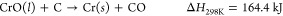
2
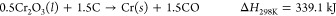
3For both reactions, Cr-oxide species are assumed to be in liquid form, and Cr metal is a BCC type alloy. Thermodynamic simulations with CrO (*l*) as the reactant predict the formation of M_7_C_3_ at a lower temperature than it is with Cr_2_O_3_ (*l*). For instance, Cr_2.5_Fe_4.5_C_3_ forms from CrO (*l*) while there is only trace Cr in FCC in the case of Cr_2_O_3_ (*l*) at 1000 °C with the formation of M_7_C_3_ from Cr_2_O_3_ (*l*) being delayed to 1200 °C.

Consumption of the dissolved species of Fe and Cr on carbon/alloy particles would be the driving force behind the transport or diffusion of the species in molten slag/salt. The reaction at the reduction site must be faster than the chromite dissolution reaction to keep the molten slag/salt undersaturated with respect to Fe and Cr. If the transport is not fast enough due to the viscosity of the melt or the reduction reactions are slow, the melt can reach saturation with respect to Fe and Cr species, which would result in the formation of Fe–O and/or Cr–O compounds. As the alloy rim grows at the expense of the shrinking carbon particle, diffusion of species across the alloy could become the rate-limiting step, especially toward the final stages as the alloy rim thickens. The reaction kinetics discussed earlier indicates that the overall reaction can be illustrated topologically as shrinking cores of petcoke particles as the reaction progresses (Figure 2). The overall reduction reaction can be represented by reaction 4 where the reactant and product compositions are those indicated earlier in the Materials and methods section.
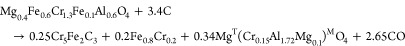
4where the superscript T represents tetrahedral and M stands for octahedral sites in the spinel or residual chromite crystal structure.

In this case, one mol chromite reacts with 3.4 mol C to form a M_7_C_3_ type carbide, Cr-austenite, spinel, and CO. There are slight imbalances of Cr, Fe, Mg and Al in the products that amount to 0.04 excess Cr, and deficiencies of 0.02 Fe, 0.03 Mg and 0.02 Al. These are minor which are likely to be within uncertainties of the average compositions assumed for the reactants and products. In addition, the inclusion of clinochlore in the reactants and aluminosilicate slag in the products would further refine the stoichiometry of the overall reaction.

## 4 Conclusions

Majority of Fe losses from the crystal structure of chromite during its incongruent dissolution occurred when 1100 °C is reached. At 1150 °C, all Fe^2+^ is lost, and about one-half of Cr remains in chromite. After 2 h of reaction at 1300 °C, nearly all Cr is dissolved with the resultant composition of Mg_1.0–1.2_Al_1.7–1.9_Cr_0.0–0.1_O_4_. Following the onset of Cr reduction at 1100 °C, changes in the alloy composition from Cr_4.1_Fe_2.9_C_3_ to Cr_5.9_Fe_1.1_C_3_ between 1200 and 1300 °C also reflect the switch to Cr dominant reduction. These residual chromite and alloy compositions are consistent with the predicted equilibrium compositions, indicating that near equilibrium conditions are reached after 2 h of reaction at 1300 °C.

After 15 min of reaction at 1300 °C, the Cr/Fe mass ratios of ferrochrome reaches the 2.1–3.0 range which covers the Cr/Fe ratio of the ore in the feed (i.e., 2.1–2.2), suggesting that the alloy composition evolves fast with the reduction and transport of Cr species while reaching its near-equilibrium value before the bulk of Cr in the ore is reduced. After 2 h of reactions at 1300 °C, more than 95% Cr metallization is achieved.

Slag melt forming at 800 °C results from congruent dissolution of clinochlore and it is saturated with respect to forsterite. Chromite dissolution begins at 950 °C as evidenced from rimmed chromite particles. Slags formed between 1200 and 1300 °C are compositionally similar. Increased levels of Cr concentrations in the interstitial slag are noted to occur at 1200 °C, coinciding with the abundance of chromite particles with reaction rims. Chromium concentrations in interstitial slag, which are stabilized at 0.3 to 0.9 wt % after 15 min of reduction at 1300 °C, would be well below the solubility limits of slag characteristic of the DRC process. This is because chromite dissolution and Cr reduction reactions are occurring in unison, controlling the Cr concentrations through the solubility and transport of Cr enabled by the Cl-rich slag/salt media.

Four types of reduced Cr species were identified in the slags and residual chromite. Type 1 representing slags that formed at 1300 °C after 3 h of reaction is made of Cr^2+^ species that are coordinated to oxygen. Type 2 is made of Cr^2+^ species mixed with minor Cr^3+^. It is typically observed in monticellite formed after 4 h at 1300 °C, containing 1.94 ± 0.52 wt % Cr as Cr_2_O_3_ and occurring as groundmass to chromite and alloy particles. Type 2 species is also inferred to be present in various slags formed at 1200 and 1300 °C. Type 3 species are Cr^2+^ which are coordinated to chlorine and oxygen. It is typically observed in residual chromite rims with pores of slag melt and in a Ca–Mg aluminosilicate slag with Cl that formed at 1100 °C. Type 3 species forms the most abundant reduced Cr species present in the products representing the experimental range from 1100 to 1300 °C. The presence of Type 3 species in both chromite rims and slag suggests that chromite rims equilibrated with a slag melt containing reduced Cr species or the XANES spectra represent small slag pockets in residual spinel. Type 4 represents the most reduced species with similarities to zerovalent chromium. Type 4 is observed in a Ca–Si–Cl slag that formed at 1200 °C.

Discovery of reduced Cr species in slag and other phases indicates that reduction of Cr from 3+ to 2+ and 0 occurred immediately after the dissolution of Cr_2_O_3_ to slag/salt melt prior to their arrival at solid carbon particles. EXAFS fitting of Type 1 slag indicated that Cr^2+^ is coordinated to 5 oxygen and 1 chlorine atoms at radial distances of 2.01 and 2.68 Å, respectively. The absence of higher shells in EXAFS spectra points to the occurrence of Cr(O,Cl) as monomeric species in molten slag during their transport from the dissolution to the reduction sites.

The DRC process can be described as the shrinking cores of chromite and carbon particles occurring in unison involving incongruent dissolution of chromite in molten CaCl_2_ followed by reduction of Cr in slag melt, transport of Cr and Fe species in molten media and final reduction on solid carbon particles and growth as an M_7_C_3_ type carbide.

These findings combined with the consideration that reduction of CrO(*l*) to Cr metal is thermodynamically feasible at a lower temperature than it is for with Cr_2_O_3_(*l*), underscore the accelerated reduction efficiency of the CaCl_2_-assisted DRC process.

## Supporting Information Available

Additional experimental data including Cr *K*-edge micro-XANES spectra with least-squares fits, photomicrographs of experimental run products, quantitative X-ray maps and synchrotron XRF maps (PDF)
