# Supplementary material for: Mechanism of the Direct Reduction of Chromite Process as a Clean Ferrochrome Technology
Source: ACS Eng Au. 2023 Dec 1;4(1):125–38. doi: 10.1021/acsengineeringau.3c00057 (PMC10885147; doi:10.1021/acsengineeringau.3c00057)
Supplement: Supplementary file 1 — eg3c00057_si_001.pdf [file eg3c00057_si_001.pdf]

Mechanism of the Direct Reduction of Chromite Process as a Clean Ferrochrome Technology

Supplementary Information

Dogan Paktunc*, Jason P. Coumans, David Carter, Nail Zagrtdenov, Dominique Duguay

CanmetMINING, 555 Booth Street, Ottawa, ON, K1A 0G1

*Corresponding author dogan.paktunc@nrcan-rncan.gc.ca

Table S1. Composition of the chromite ore used in the experiments and average compositions of chromite and clinochlore as the dominant gangue mineral in the Ring of Fire chromite ores

	Chromite Ore	Chromite	Clinochlore
SiO_2	6.46±2.46	nd	32.01±1.80
TiO_2	0.33±0.03	0.56±0.26	0.01±0.01
Al_2O_3	12.08±1.08	14.01±2.53	14.83±2.73
Cr_2O_3	42.31±3.32	49.49±3.33	3.62±2.02
FeO(t)	18.85±1.93	26.75±5.95	1.43±0.44
CaO	nd	nd	0.02±0.02
MgO	14.88±1.59	7.82±1.96	35.15±1.29

Ore represents the -106+75 µm size fraction of the Black Horse chromite ore from the Ring of Fire chromite deposits. Chromite represents more than 4300 and clinochlore more than 125 electron probe microprobe analyses (Laarman 2014[13] and this study); nd: not determined

Table S2. Slag compositions (wt%) formed at various reaction times at 800 to 1300 °C

	800-15 (n=10)		950-15 (n=21		1000-5 (n=6)	
	mean±SD	range	mean±SD	range	mean±SD	range
SiO_2	30.64±3.86	23.74-34.83	26.75±1.34	23.80-29.21	28.37±2.05	25.33-30.91
TiO_2	0.07±0.03	0.04-0.11	0.12±0.04	0.04-0.19	0.09±0.07	0.01-0.17
Al_2O_3	16.76±2.76	13.99-21.84	17.63±5.43	11.76-28.09	18.74±5.65	11.47-23.65
Cr_2O_3	2.60±0.81	0.88-3.30	2.02±1.23	0.79-5.93	1.25±1.00	0.57-3.21
FeO	0.77±0.17	0.41-0.97	0.52±0.41	0.19-1.96	0.26±0.10	0.13-0.37
MgO	24.47±8.45	11.01-36.63	9.66±3.56	3.46-16.71	9.31±1.79	6.36-11.23
CaO	15.84±5.96	7.04-25.38	33.35±4.86	23.26-40.31	30.02±6.79	22.14-38.28
Cl	7.65±4.06	1.58-13.81	12.57±1.40	10.31-15.34	12.60±1.08	11.46-14.53

	1100-5 (n=9)		1200-5 (n=8)		1200-30 (n=11)	
	mean±SD	range	mean±SD	range	mean±SD	range
SiO_2	26.01±1.02	24.30-27.33	29.12±1.12	26.85-30.36	32.63±8.67	23.63-46.23
TiO_2	0.14±0.05	0.07-0.23	0.63±0.23	0.26-1.06	0.43±0.25	0.07-0.79
Al_2O_3	16.70±1.98	15.02-20.17	8.53±0.67	7.62-9.56	8.74±4.17	4.66-18.27
Cr_2O_3	1.02±0.46	0.43-1.66	1.48±1.18	0.51-3.34	1.62±1.63	0.42-5.29
FeO	0.21±0.10	0.08-0.36	0.24±0.26	0.10-0.87	0.13±0.04	0.08-0.21
MgO	8.91±2.57	6.36-13.59	11.50±1.61	9.89-14.20	9.09±3.71	3.59-13.76
CaO	38.26±2.25	34.19-40.29	39.57±2.16	36.60-42.17	38.60±4.09	31.85-43.44
Cl	12.63±0.80	11.21-13.39	12.70±0.73	11.79-13.55	12.88±1.07	10.36-13.74

	1300-1 (n=8)		1300-15 (n=7)		*1300 (n=23)	
	mean±SD	range	mean±SD	range	mean±SD	range
SiO_2	27.57±2.84	22.32-30.33	28.26±0.72	27.39-29.29	36.33±0.32	35.75-36.82
TiO_2	0.69±0.11	0.52-0.81	1.24±0.20	0.94-1.49	1.09±0.03	1.05-1.17
Al_2O_3	8.72±2.95	5.85-14.35	7.76±1.04	6.33-9.41	18.84±0.81	17.39-20.06
Cr_2O_3	3.50±3.48	0.38-8.97	0.67±0.21	0.30-0.90	3.46±0.30	3.01-4.10
FeO	0.38±0.52	0.10-1.65	0.08±0.02	0.05-0.11	0.06±0.03	0.03-0.15
MgO	11.59±1.40	10.35-14.43	10.89±0.12	10.72-11.03	1.58±0.26	1.02-2.09
CaO	38.56±4.30	31.32-42.69	41.43±0.81	40.42-42.72	34.28±0.61	33.32-35.36
Cl	12.32±1.40	9.91-13.67	12.91±0.34	12.35-13.34	4.04±0.18	3.71-4.40

Numbers before the dash are temperature in Celsius and those following are reaction times in minutes. n: number of analyses (each representing 3 sigma uncertainty). SD: standard deviation. *1300: slag formed from feed with 4.7% bentonite after 3 hours of reaction.

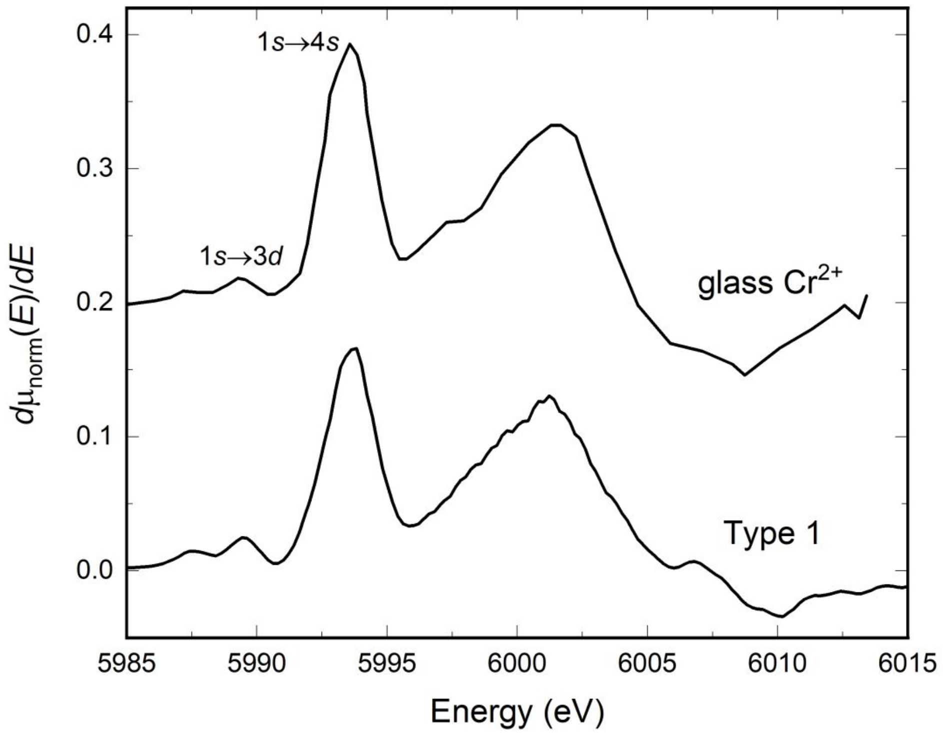

Figure S1. Derivative Cr K-edge XANES spectrum of Type 1 slag shown in comparison to that of Cr^{2+} glass of Berry and O'Neill (2004) (digitized spectrum shifted 1.5 eV to lower energies to line up with the 1*s*→3*d* peak of the slag). Type 1 slag has 1*s*→3*d*, 1*s*→4*s* and the main peaks at 5989.5, 5993.7 and 6001.2 eV, respectively.

Table S3 – Summary of Cr species present in experimental run products

exp#	number of XANES scans	Type 1	Type 2	Type 3	Type 4
		Cr^{2+}	Cr^{2+} Cr^{3+}	Cr^{2+}	Cr^{0}
4	6			+	
5	10			+	+
6	7		+	+	+
7	84	+	+	+	+
8	30	+	+	+	
9	41		+	+	+
10	16			+	+
11	114	+	+	+	+
12	20	+			

Refer to Table 1 in the manuscript for an explanation of the experiments (exp#).

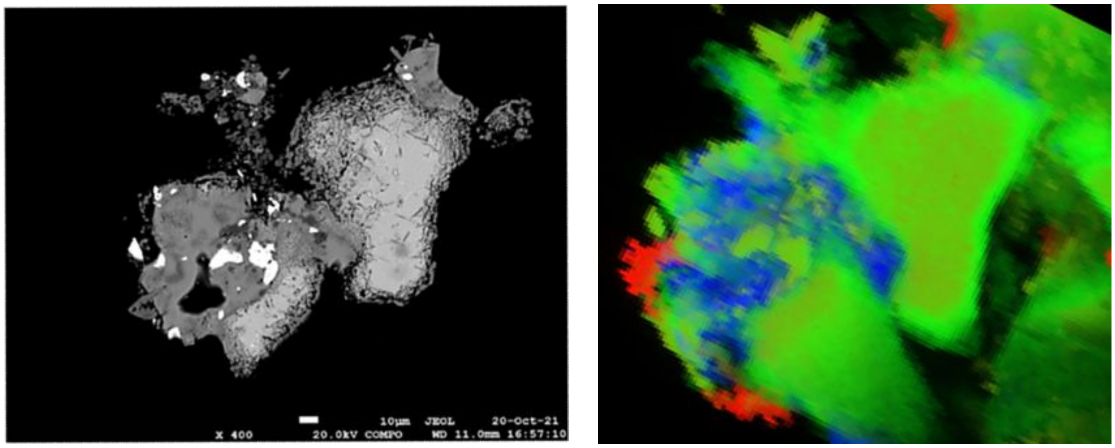

Figure S2. BSE image and corresponding sXRF map showing interstitial slag (dark grey; blue) and residual and partially dissolved chromite particles (grey; green with orange tint) with reaction rims (dark grey; green) formed at 1200 °C after 30 minutes (Experiment 7). Micro-XANES spectra indicate that chromite rims are composed of 70% chromite (Cr^{3+}), 17% Type 3, 12% Type 1 and 10% Type 2. Slag is made of Cr^{3+}, Type 2 and Type 3 species.

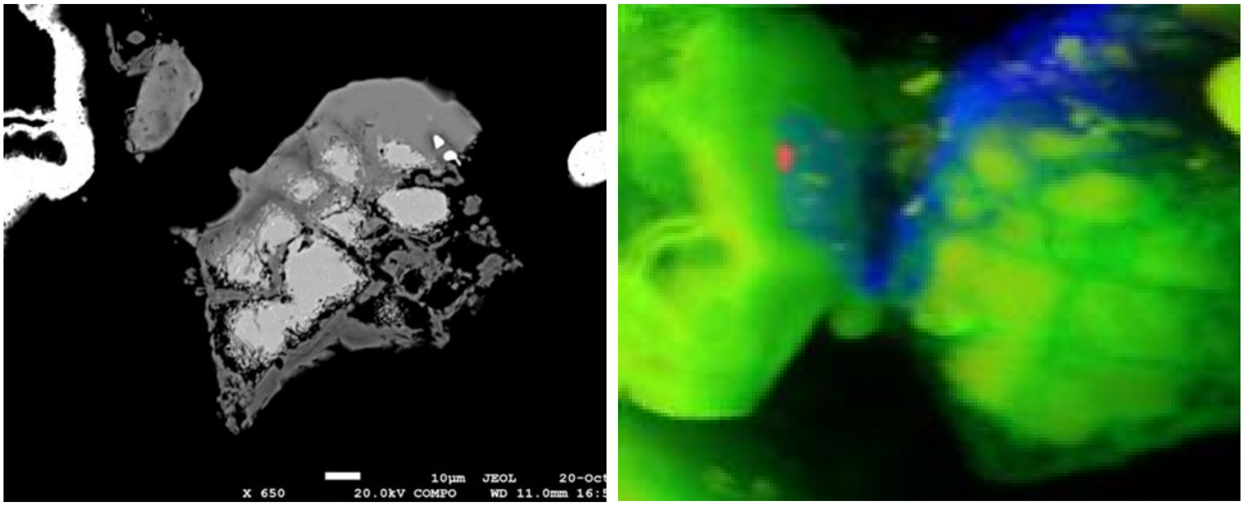

Figure S3. BSE image and corresponding sXRF map showing residual chromite (light grey; green with orange tint) enveloped by slag (dark grey; blue) formed after 30 minutes of reaction at 1200 °C (Experiment 7). Alloy is white on the BSE image and brownish green on the sXRF image. Micro-XANES spectra from interstitial slag areas (blue) indicate mixed species composed of 41% Type 3, 33% Type 2 and 21% chromite.

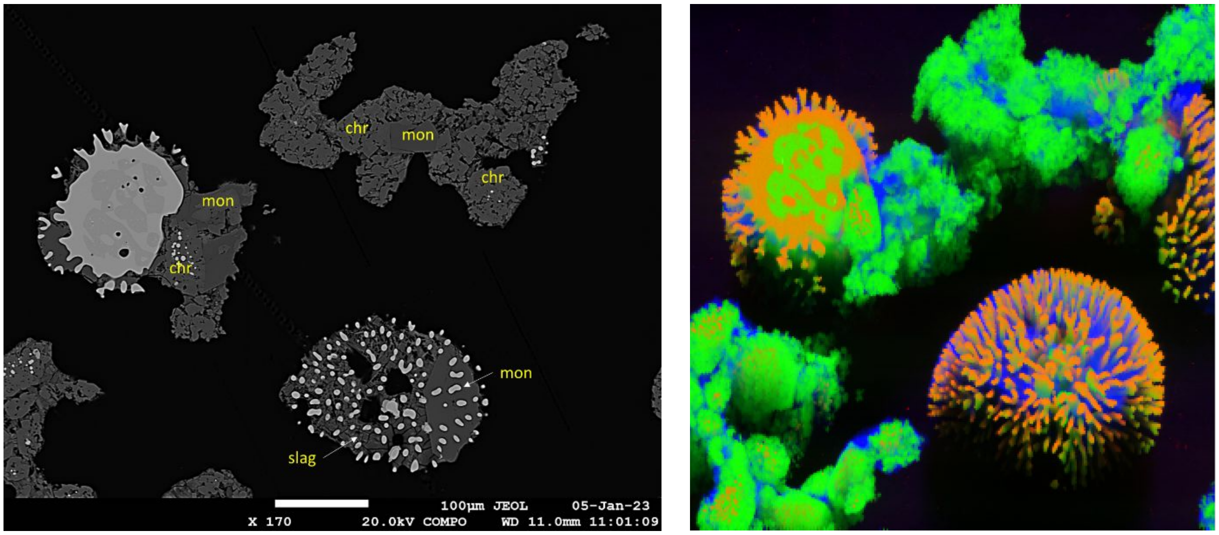

Figure S4. BSE image and corresponding sXRF map of slag (grey/blue), monticellite (mon) (grey/blue) and residual chromite (chr) (grey/green) representing a product where supersaturation of the slag is promoted using sub-stoichiometric carbon in the feed. Sponge-looking spherical particle with FeCr stringers (orange) form an interconnected network likely to be resulting from supersaturation of reduced Cr and Fe species in melt. The interstitial slag forming matrix of the sponge-looking spherical particle is dominated by Cr^0 as represented by Type 4 and FeCr with minor Cr^{3+} as represented by chromite. Monticellite intermingled with FeCr stringers is dominated by Cr^{2+} as represented by Type 2 with a smaller proportion of Cr^0 as represented by Type 4. Chromite which is $MgAl_{1.0\text{-}1.1}Cr_{0.8\text{-}0.9}Mg_{0.1}O_4$ are dominated by Cr^{2+} (Type 3) with smaller proportions of Cr^{3+}.

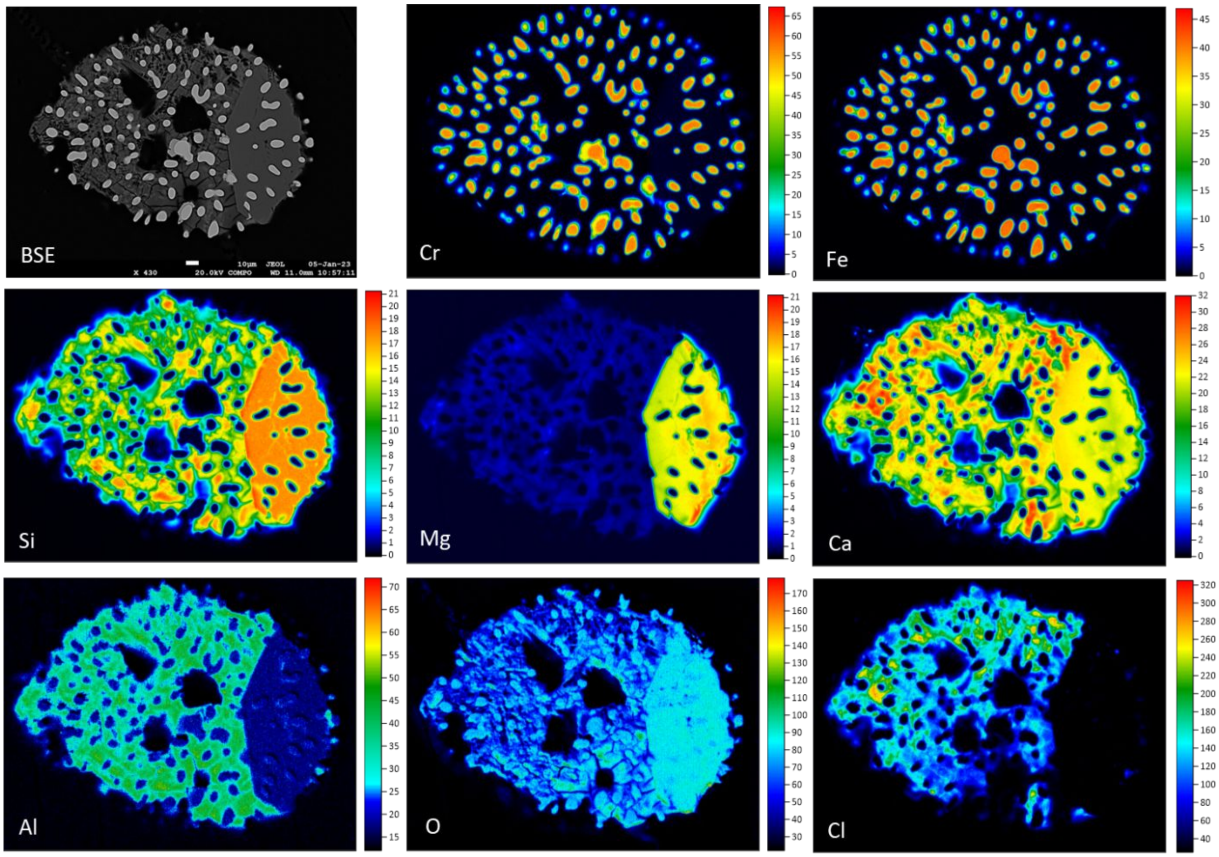

Figure S5. BSE image and corresponding quantitative X-ray maps of the sponge-looking spherical particle in Figure S4 showing compositional variation of the interstitial slag dominated by Cr^0 species. Monticellite forms the matrix on the right side that is enriched in Mg, Si and O, and depleted in Cl and Al is monticellite.

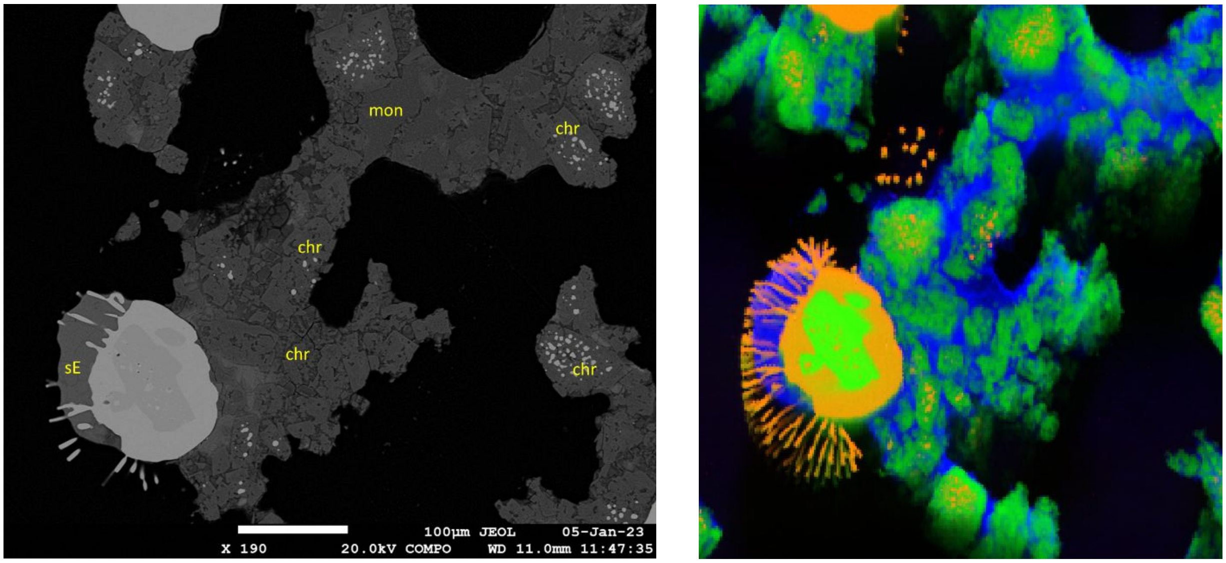

Figure S6. BSE image and corresponding sXRF map showing a FeCr particle (light grey; orange with green core) with stringers (light grey; orange) growing into the surrounding interstitial Ca-Si-Cl-Mg-Al slag (sE) (Slag E in Table 3). The slag is Type 4 mixed with FeCr. Other interstitial slag areas contain Type 3 and Type 4 species mixed with Cr^{3+} (chromite). Residual chromite (chr) has the composition of $MgAl_{1.2}Cr_{0.7}Mg_{0.1}O_4$ and it is dominated by Type 3 with minor Cr^{3+}. Monticellite (mon) ($Ca_{0.9}Mg_{1.1}SiO_4$) has Type 2 and Type 3 in addition to Cr^{3+}. Alloy core is Cr-rich (dark grey/green) having the composition of $Cr_{6.5}Fe_{0.5}C_3$. It is overgrown with stringers (light grey/orange) having a BCC-type alloy (Cr_3Fe_2).

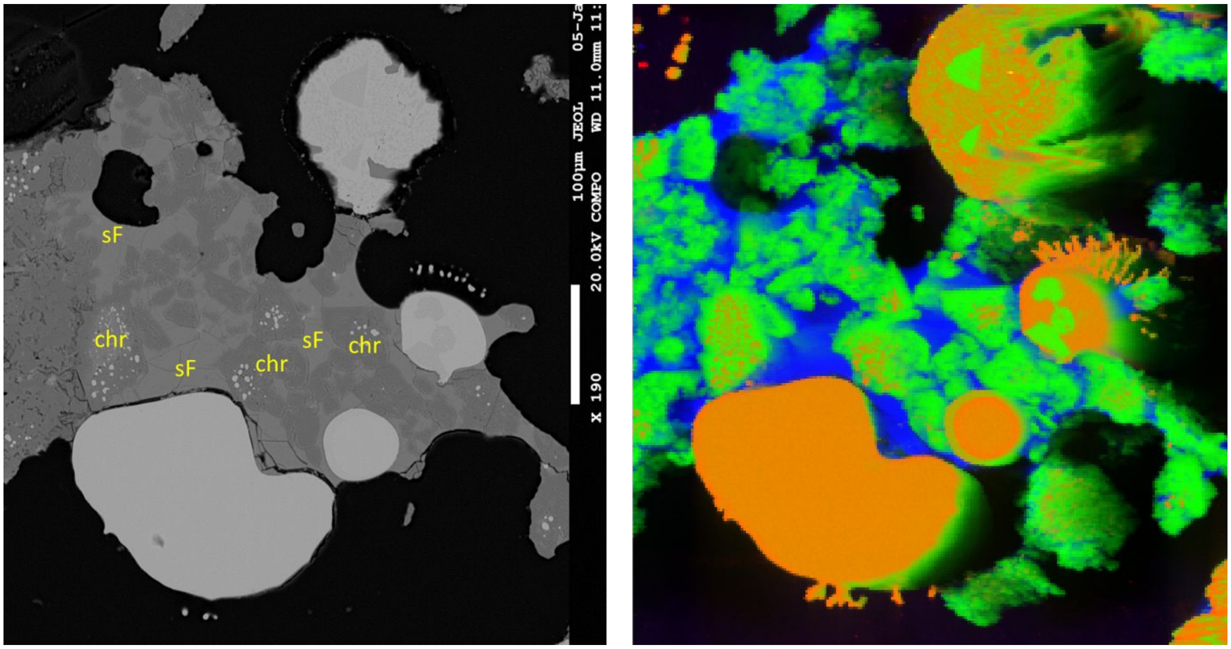

Figure S7. BSE image and corresponding sXRF map showing an interstitial Ca-Si-Cl-Mg-Al-Cr slag (sF) (Slag F in Table 3) made of Type 3 (15-32%), Type 1 (<33%), chr (23-55%) and Type 4 (0<12%) Cr species. Chromite (chr) which is $MgAl_{1.2}Cr_{0.7}Mg_{0.1}O_4$ is dominated by Type 3 (62-95%), chromite (4-13%) and Type 2 (7-16%).

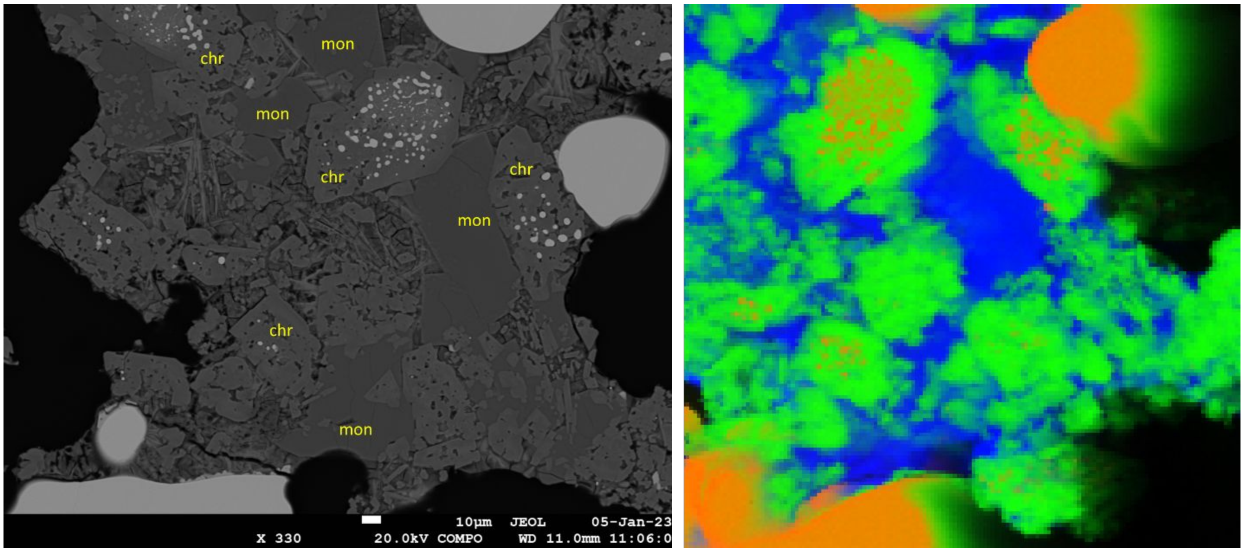

Figure S8. BSE image and corresponding sXRF map showing monticellite (mon/blue) ($Ca_{0.9}Mg_{1.1}SiO_4$) having Type 2 Cr species. It can also contain up to ~15% Type 1 and ~10% Cr^{3+} species. Chromite (chr) which has the composition of $MgAlCr_{0.9}Mg_{0.1}O_4$ has 50-70% Type 3 and 30-50% Cr^{3+}.

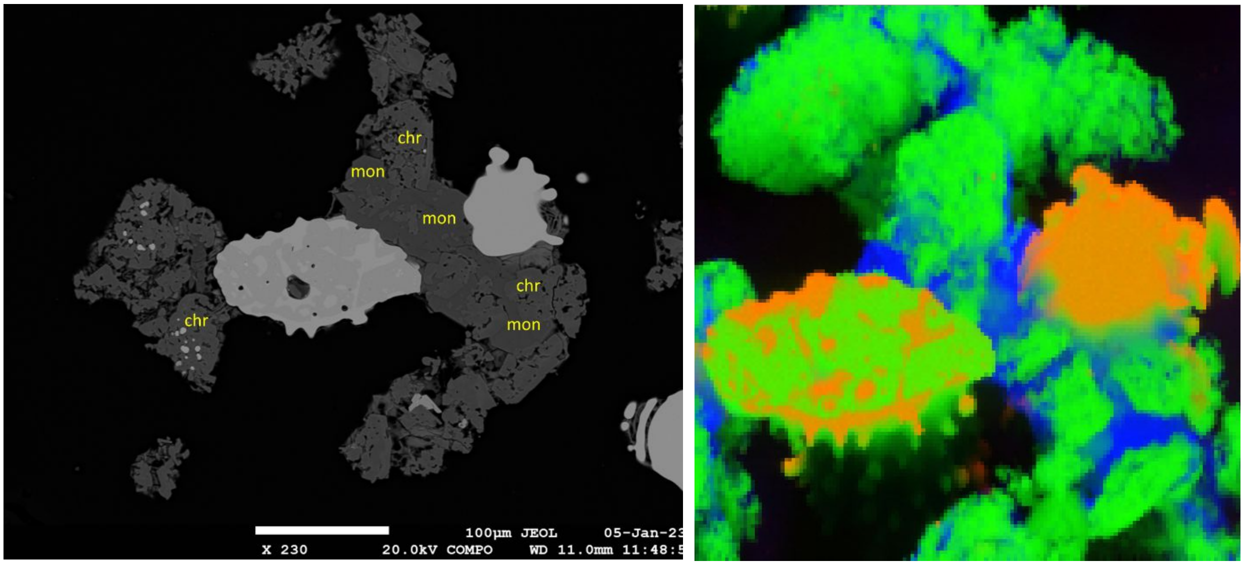

Figure S9. BSE image and corresponding sXRF map showing monticellite (mon/blue) ($Mg_{1.1}Ca_{0.9}SiO_4$) formed from slag surrounding alloy (light grey/orange-green) and residual chromite (dark grey/green) particles. Monticellite is made of 70-100% Type 2 and <30% Cr^{3+}. Residual chromite (chr) which is $MgAl_{1.0\text{-}1.2}Cr_{0.7\text{-}0.8}Mg_{0.1}O_4$ is made of 30-70% Type 3 and 30-70% Cr^{3+}.

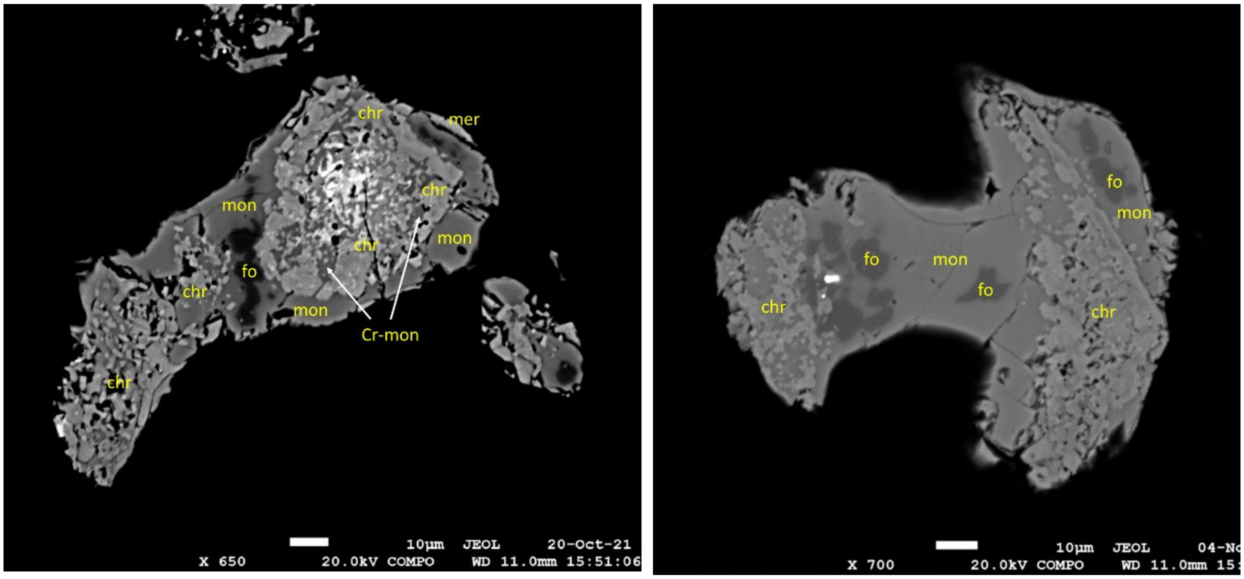

Figure S10. BSE images of incongruently dissolving chromite with slag melt in small pockets/pores which has the composition of Cr-bearing monticellite. Residual chromite is surrounded by monticellite and merwinite. Bulk slag surrounding chromite is monticellite as the slag. Chromite in core is $MgAl_{0.5}Cr_{1.2}Fe_{0.3}O_4$ whereas chromite on the rim is $MgAlCr_{0.9}Mg_{0.1}O_4$. It appears from the photograph on the right that forsterite is the last phase precipitating from the residual slag melt pockets following monticellite formation

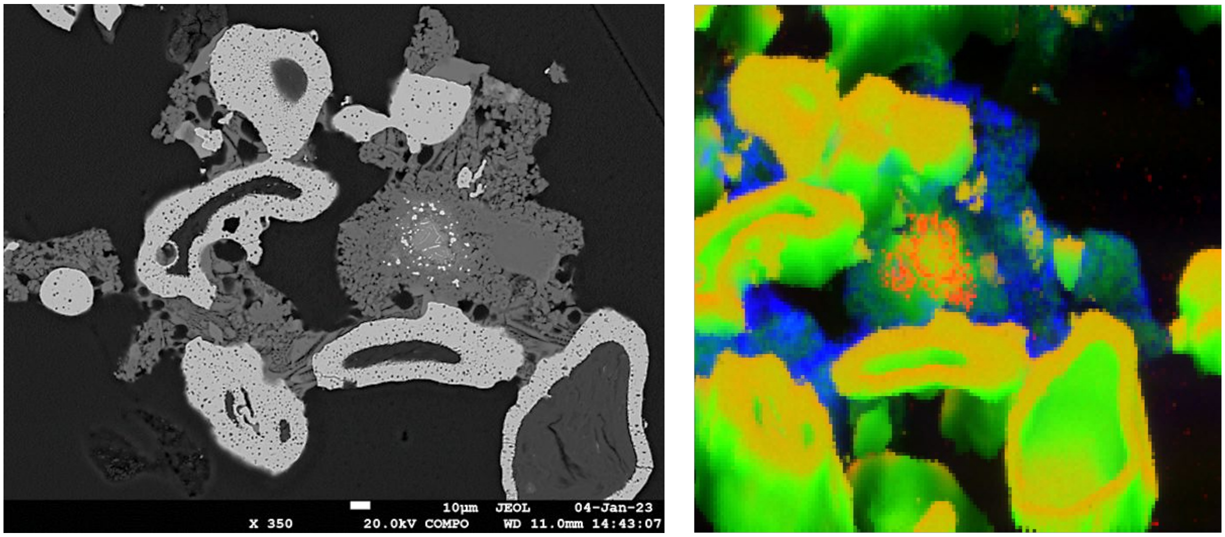

Figure S11. BSE image and corresponding sXRF map of slag (blue) and residual chromite (green) areas representing Experiment 9 conducted to assess rate control by diffusion of species across growing alloy rim over carbon particle at 1300 °C for 15 minutes. Orange areas represent FeCr alloy whereas small red particles in residual chromite represent Fe-rich BCC. The slag possesses Type 3 species mixed with Cr^0 (FeCr and/or Type 4) and Cr^{3+} as in chromite.

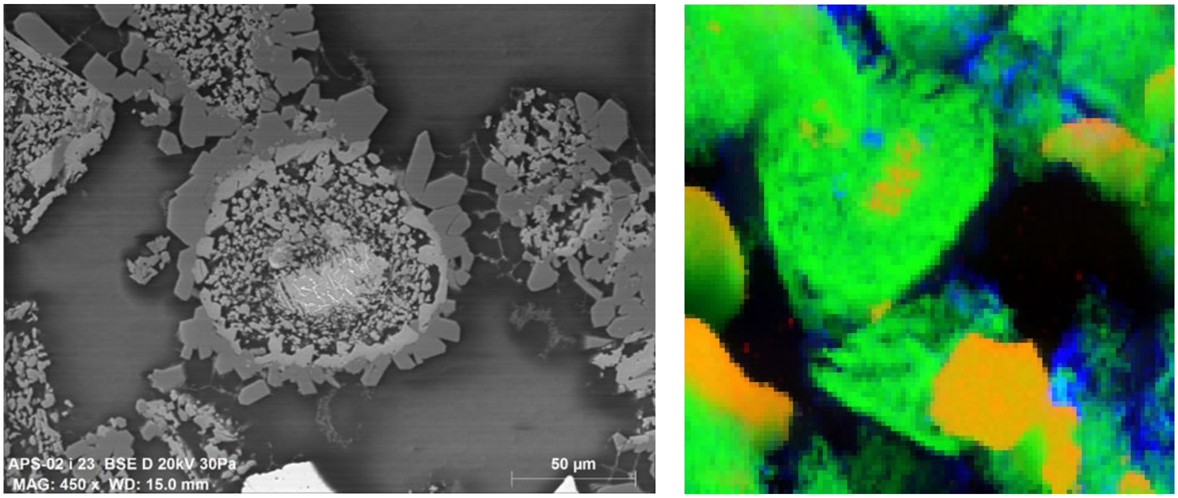

Figure S12. BSE image and corresponding sXRF map of a zoned chromite (light grey/green) rimmed by forsterite (medium grey) formed after 15 minutes at 1300 °C (Experiment 9). Chromite rim which is $MgCr_{0.7}Al_{1.2}Mg_{0.1}O_4$ possesses Type 3 mixed with Cr^{3+} and Type 2. Forsterite is made of 65-70% Type 3, 15% Type 2 and 15% Cr^{3+}.

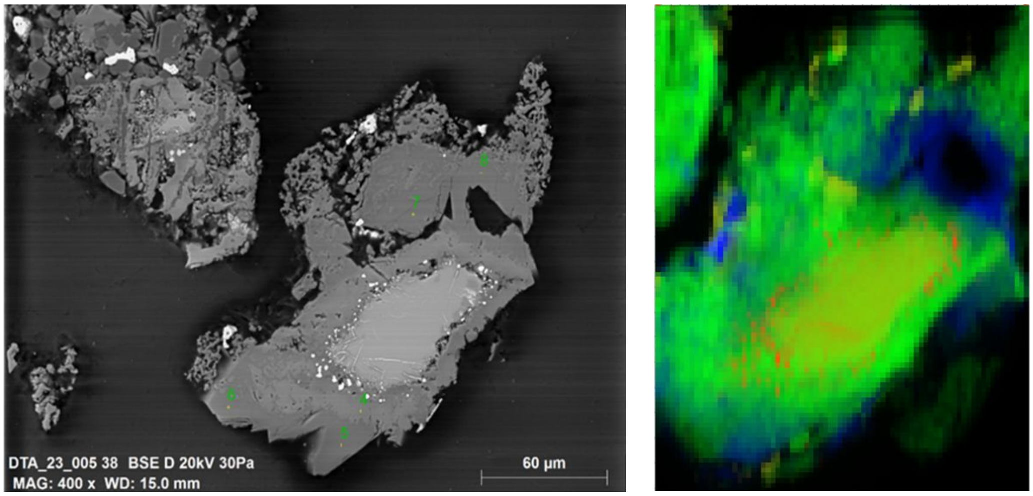

Figure S13. BSE image and corresponding sXRF map of a shrinking core of chromite (light grey; brownish green) surrounded by Cr-poor spinel (grey; green) and interstitial slag (grey; blue) formed at 1300 C after 15 minutes (Experiment 9). Chromite rim possesses Type 3 species mixed with Cr^{3+}. Slag which is a Ca-Mg aluminosilicate with minor Cr has Type 2 and Type 3 Cr species.

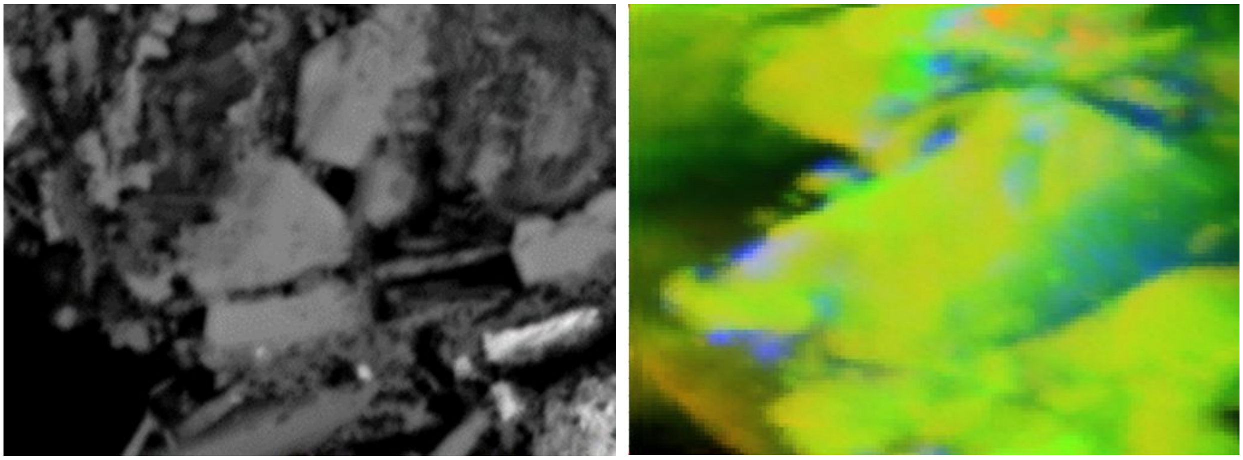

Figure S14. BSE image and corresponding sXRF map of a wadalite-like slag (medium grey; blue) possessing Type 3 species in the experimental run product formed at 1100 °C after 5 minutes of reaction (Experiment 4) signifying the onset of Cr reduction. Width of the BSE image is 80 μm and the sXRF map is 30 μm.

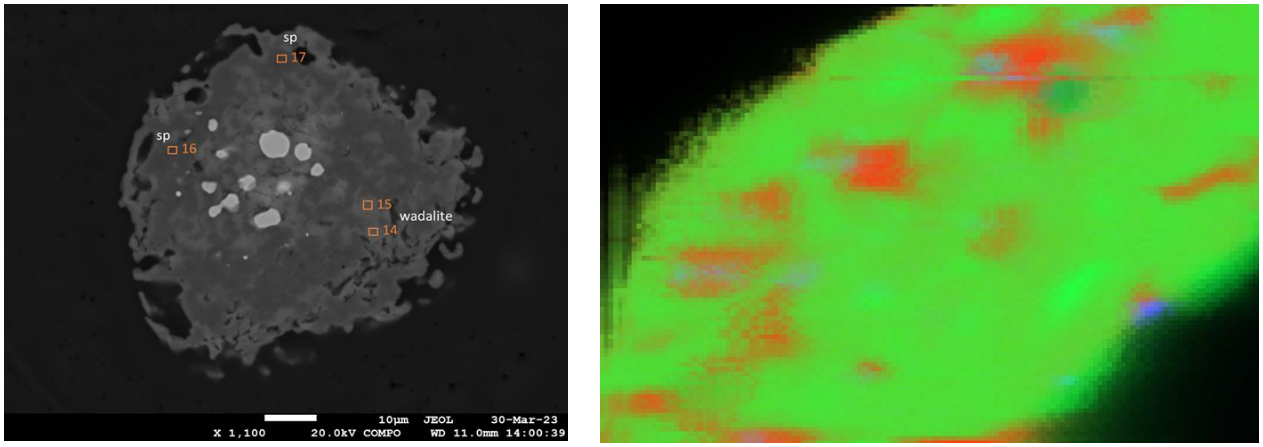

Figure S15. BSE image and corresponding sXRF map of wadalite-like slag (dark grey) with FeCr alloy grains (light grey), representing a fully reduced product formed at 1300 °C after 2 hours (Experiment 10). Micro-XANES spectra indicate that the slag has about 85 % Cr^0 as represented by 55 % FeCr and 30 % Type 4, with the reminder being made of Type 3 species.

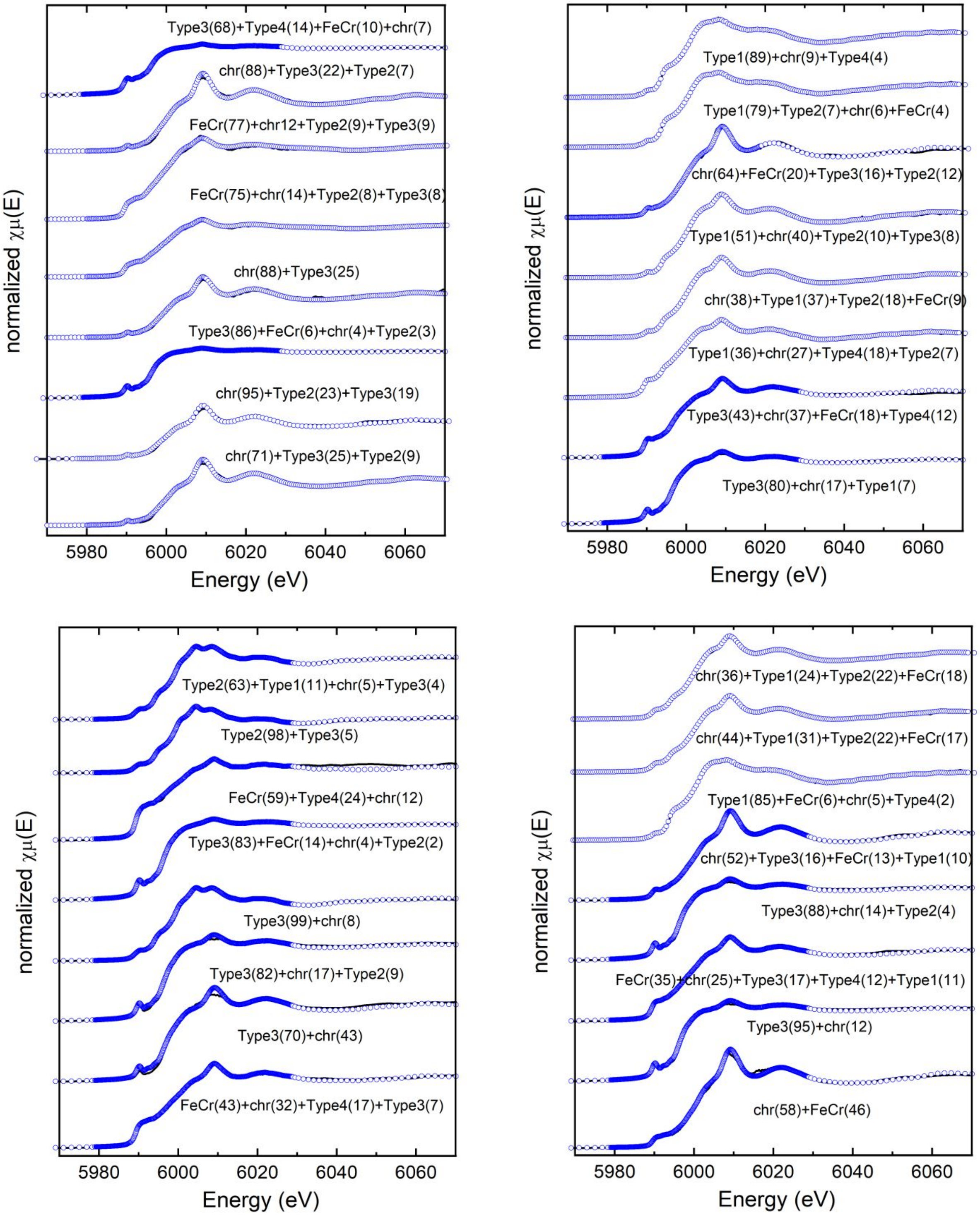

Figure S16. Representative examples of least squares fitting of Cr *K*-edge XANES spectra with end members of chromite (chr), M_7C_3-type ferrochrome (FeCr), Type1, Type2, Type3 and Type 4 compounds. Measured spectra shown in solid black lines behind the fitted spectra shown in blue circles. Proportions of end members are given in brackets. Sum of the quantities not forced to 100% during fitting to allow for assessing fit quality with an additional parameter.
